# Genome‐environment association analyses reveal geographically restricted adaptive divergence across the range of the widespread Eurasian carnivore *Lynx lynx* (Linnaeus, 1758)

**DOI:** 10.1111/eva.13570

**Published:** 2023-10-09

**Authors:** Enrico Bazzicalupo, Mirosław Ratkiewicz, Ivan V. Seryodkin, Innokentiy Okhlopkov, Naranbaatar Galsandorj, Yuriy A. Yarovenko, Janis Ozolins, Alexander P. Saveljev, Dime Melovski, Alexander Gavashelishvili, Krzysztof Schmidt, José A. Godoy

**Affiliations:** ^1^ Department of Ecology and Evolution Estación Biológica de Doñana (CSIC) Seville Spain; ^2^ Faculty of Biology University of Białystok Białystok Poland; ^3^ Laboratory of Ecology and Conservation of Animals Pacific Institute of Geography of Far East Branch of Russian Academy of Sciences Vladivostok Russia; ^4^ Institute for Biological Problems of Cryolithozone Siberian Branch of the Russian Academy of Sciences Yakutsk Russia; ^5^ Institute of Biology Mongolian Academy of Sciences Ulaanbaatar Mongolia; ^6^ Pre‐Caspian Institute of Biological Resources Dagestan Federal Scientific Centre of RAS Makhachkala Russia; ^7^ Department of Hunting and Wildlife Management Latvijas Valsts mežzinātnes institūts "Silava" Salaspils Latvia; ^8^ Department of Animal Ecology Russian Research Institute of Game Management and Fur Farming Kirov Russia; ^9^ Macedonian Ecological Society (MES) Skopje North Macedonia; ^10^ Center of Biodiversity Studies, Institute of Ecology Ilia State University Tbilisi Georgia; ^11^ Mammal Research Institute Polish Academy of Sciences Białowieża Poland

**Keywords:** conservation units, Eurasian lynx, genome scans, local adaptation, *Lynx lynx*, subspecies

## Abstract

Local adaptations to the environment are an important aspect of the diversity of a species and their discovery, description and quantification has important implications for the fields of taxonomy, evolutionary and conservation biology. In this study, we scan genomes from several populations across the distributional range of the Eurasian lynx, with the objective of finding genomic windows under positive selection which may underlie local adaptations to different environments. A total of 394 genomic windows are found to be associated to local environmental conditions, and they are enriched for genes involved in metabolism, behaviour, synaptic organization and neural development. Adaptive genetic structure, reconstructed from SNPs in candidate windows, is considerably different than the neutral genetic structure of the species. A widespread adaptively homogeneous group is recovered occupying areas of harsher snow and temperature climatic conditions in the north‐western, central and eastern parts of the distribution. Adaptively divergent populations are recovered in the westernmost part of the range, especially within the Baltic population, but also predicted for different patches in the western and southern part of the range, associated with different snow and temperature regimes. Adaptive differentiation driven by climate does not correlate much with the subspecies taxonomic delimitations, suggesting that subspecific divergences are mostly driven by neutral processes of genetic drift and gene flow. Our results will aid the selection of source populations for assisted gene flow or genetic rescue programs by identifying what climatic patterns to look for as predictors of pre‐adaptation of individuals. Particularly, the Carpathian population is confirmed as the best source of individuals for the genetic rescue of the endangered, isolated and genetically eroded Balkan population. Additionally, reintroductions in central and western Europe, currently based mostly on Carpathian lynxes, could consider the Baltic population as an additional source to increase adaptive variation and likely improve adaptation to their milder climate.

## 1 INTRODUCTION

Local adaptations are expected to shape species diversity and contribute to intraspecific divergence and may eventually lead to speciation (de Queiroz, 2007). The identification of locally adapted populations should thus play a key role in the delimitation of evolutionary significant units (ESUs) for species conservation (Funk et al., 2012; Ryder, 1986), designation of ecotypes (Stronen et al., 2022) and subspecies (Schmidt et al., 2019). While there are still some aspects of the significance of adaptive genetic variation that remain not fully grasped, it is widely recognized that it plays an important role in speciation pathways and the global distribution of organisms (Doebeli & Dieckmann, 2004; Weissing et al., 2011). Understanding and describing species‐wide patterns of functional genetic diversity is therefore key to prioritizing conservation efforts and guiding taxonomic delimitation (Schmidt et al., 2019; Stronen et al., 2022; Teixeira & Huber, 2021).

One approach to predict and quantify adaptive population structure is trying to locate the areas of the genome which have been subjected to differential selective pressures among populations. Different approaches have been developed to achieve this goal (Hoban et al., 2016). Many studies have focused on the identification of genomic regions maximally differentiated among populations through summary statistics of genetic differentiation measured along the genome (Duforet‐Frebourg et al., 2014; Gautier, 2015). One limitation of this type of inference is the inability to identify the underlying environmental or ecological factors driving the observed differentiation, beyond postulations based on the function of the genes involved. Other approaches have tried to work around this limitation by assessing directly the correlation between potential predictors (e.g. environmental variables) and genetic variation. These methods are commonly known as genome‐environment association (GEA) analyses, and may be based on univariate and multivariate approaches, or a combination of both, which provides a higher power of detection (Forester et al., 2018). Loci identified as associated to local adaptation by any method will likely show a pattern of genetic structure different to that observed at neutral loci. Whereas the former will reflect the gradients of adaptive pressures (Nosil et al., 2008; Wang & Bradburd, 2014), the latter will be determined by the interaction of genetic drift and gene flow across space and time, and thus is usually correlated with geographic distance or past isolation in glacial refugia (Sobel et al., 2010; Wright, 1943).

Although long‐range dispersal was previously believed to counteract the population structuring, processes like genotype driven dispersal (Bolnick & Otto, 2013) or isolation by environment (Wang & Bradburd, 2014) may contribute to population differentiation and local adaptations. Large carnivores, in particular, often show genetic differentiation between geographically close populations, despite their ability to move long distances within their individual life‐span. For example, climate and habitat variability were found to affect both genetic and phenotypic dissimilarities among grey wolf populations (Geffen et al., 2004; Musiani et al., 2007). Schweizer et al. (2016) found correlations of functional gene variants with temperature, precipitation, and vegetation variables among wolf ecotypes in genes related to morphology, vision, metabolism, and thermoregulation. Similarly, population structure of Canadian lynx has been associated to differences in snow conditions across its distribution (Stenseth, Shabbar, et al., 2004).

The Eurasian lynx (*Lynx lynx* Linnaeus, 1758) is a large carnivore playing an essential role as a top‐chain predator which inhabits most of the Eurasian continent, with populations having contrasting past and recent demographic histories. Although according to the IUCN the global status of this felid is Least Concern and most populations are stable, the status and trends vary greatly within its European range, with some populations being classified as endangered, while the status of Asian populations remain poorly known (Breitenmoser et al., 2015). Large and relatively diverse populations inhabit the Russian part of north‐eastern Europe and most of the Asian continent, while highly fragmented populations that suffered recent demographic bottlenecks are found in Scandinavia and the Baltic states (Bazzicalupo et al., 2022; Lucena‐Perez et al., 2020). Two other autochthonous isolated populations inhabit the Carpathian and Balkan regions (Bazzicalupo et al., 2022; Lucena‐Perez et al., 2020), whereas few small reintroduced populations have been established in several areas in Central Europe using the Carpathians as their main source population, most of them exhibiting genome‐wide diversity loss (Mueller et al., 2022). Throughout its wide distribution, the Eurasian lynx occurs in a variety of ecological and climatic conditions. They inhabit a variety of climates, with extreme differences of minimum temperatures, levels of precipitation and snow cover, and habitats including mostly evergreen boreal forest, but also deciduous woodlands, alpine zones, grasslands, xeric shrublands and semi‐deserts. The species also displays a broad phenotypic variability affecting size, morphology and pelage patterns (Darul et al., 2022; Matyushkin & Vaisfeld, 2003; Schmidt et al., 2011). Based mostly on this variability, a number of distinct Eurasian lynx subspecies have been described in different parts of the species range: *L. l. lynx* in central and eastern Europe, *L. l. balcanicus* in the south‐western Balkans, *L. l. carpathicus* in the Carpathian mountains, *L. l. isabellinus* in central Asia, *L. l. wrangeli* in central and eastern Asia and *L. l. dinniki* in the Caucasian mountains and Anatolia (Kitchener et al., 2017). Nevertheless, the genetic support based on intergenic, purportedly neutral variation has been lacking or inconclusive for some of these intraspecific subunits (Bazzicalupo et al., 2022; Lucena‐Perez et al., 2020). Genetic structure based on neutral markers, although not strong, was observed between Eurasian lynx populations, both at local and continental scales (Bazzicalupo et al., 2022; Behzadi et al., 2022; Lucena‐Perez et al., 2022, 2020; Mengüllüoğlu et al., 2021; Rueness et al., 2003, 2014; Schmidt et al., 2009). The overall consensus of these studies, backed by both nuclear and mitochondrial markers, pointed to the existence of at least three major lineages within the species, one in the East (*L. l. wrangeli*), one in the Northwest (*L. l. lynx*) and one in the Southern part of the distribution (*L. l. dinniki*). Another divergent mitogenomic lineage was suggested to occur in China (*L. l. isabellinus*) pending on nuclear corroboration (Mengüllüoğlu et al., 2021). While the genetic differentiation observed within the Western part of the Eurasian lynx range has largely been attributed to recent human‐related population declines, the genetic differentiation observed between the Eastern, Western and Southern lineages is caused by genetic isolation initiated in late Pleistocene, and intensified throughout the Holocene (Bazzicalupo et al., 2022; Lucena‐Perez et al., 2020). In a first attempt to find ecological and environmental drivers of genetic differentiation, microsatellite and mtDNA differentiation was found to be related to snow cover, NAO index and diet in the western part of the Eurasian lynx range (Ratkiewicz et al., 2014), but no studies to date have tried to describe patterns of adaptive population structure at a range‐wide scale.

In this study, we apply a combination of GEA approaches with the objective of revealing genomic variation carrying possible adaptive significance for the Eurasian lynx. We aimed at identifying areas of the genome responsible for local adaptation in different populations, identifying what genes are involved in these processes, and characterizing patterns of adaptive genetic divergence. We finally discuss the relevance of adaptive population structure in the delimitation of Eurasian lynx taxonomic and conservation units, to inform assisted gene flow and genetic rescue programs.

## 2 METHODS

### 2.1 Sampling, DNA extraction, sequencing and read alignment

**FIGURE 1:**
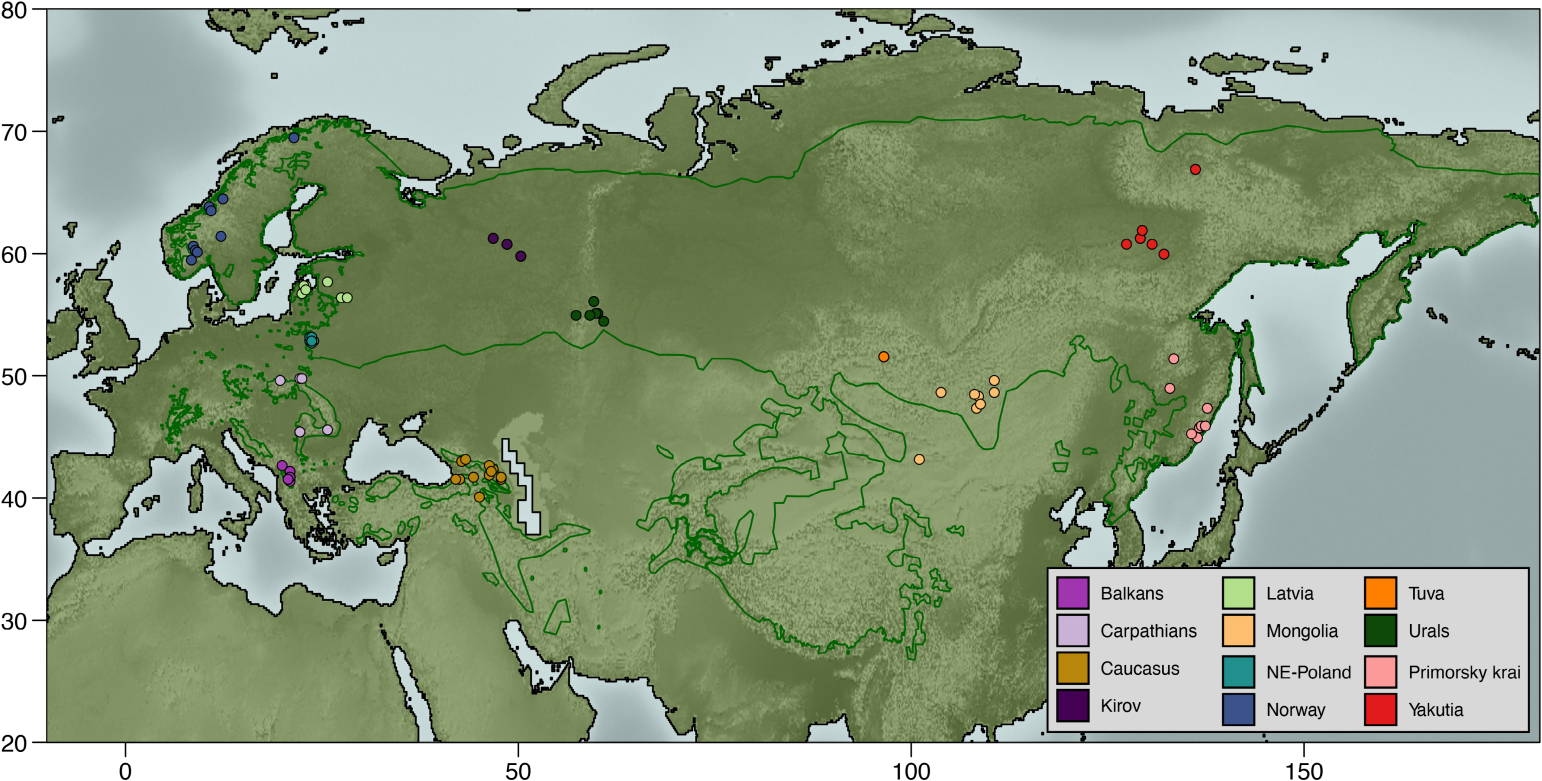
Map highlighting the distributional range of the Eurasian lynx, delimited by a green line, and locations of samples used in this study (circles, color coded by population).

We analyzed whole genome sequences of a total of 103 *Lynx lynx* individuals, comprising 12 extant populations distributed across the global range of the species and representing all proposed subspecies, except *L. l. isabellinus* (Figure 1). Most samples were tissues collected from legally hunted individuals (Norway, Latvia, Russia) or animals opportunistically found dead in the field (NE‐Poland, Carpathian Mountains, Caucasus, and Mongolia). Blood samples of live‐trapped lynx (Białowieża Primeval Forest in NE Poland and Carpathian Mountains, in Poland, and the western part of North Macedonia) were also obtained. The licenses for lynx live‐trapping and blood sampling adhere to the Directive 2010/63/EU on the protection of animals used for scientific purposes (see Bazzicalupo et al. (2022) and Lucena‐Perez et al. (2020) for details on specific license numbers). No animals were harmed during live‐trapping and handling. DNA extractions and sequencing were carried out during previous studies by our research group (Bazzicalupo et al., 2022; Lucena‐Perez et al., 2020). Samples were processed by overnight digestion using proteinase K and extracted using silica‐coated paramagnetic beads (NucleoMag® Tissue, MACHEREY‐NAGEL GmbH & Co. KG). Samples yielding DNA concentration too low to be sequenced were re‐extracted either using a classical phenol‐chloroform protocol or, in case of one Caucasian sample that was a wet claw, using a protocol meant for bone (Rohland & Hofreiter, 2007) in a sterile lab. Subsequently, gDNA was sheared, size‐selected, end‐repaired and adenylated following the appropriate Illumina protocol. After ligating indexed paired‐end adapters, DNA fragments were amplified via PCR (polymerase chain reaction) if required, and the quantity, quality and size of the libraries were assessed. Finally, libraries were sequenced using Illumina HiSeq2000 or Illumina HiSeq X‐10, in Centro Nacional de Análisis Genómico (CNAG) or Macrogen facilities, respectively. In all cases, samples were sequenced using Illumina protocols, and primary data analysis was carried out with the standard Illumina pipeline. We performed a quality control of our data using fastqc (https://www.bioinformatics.babraham.ac.uk/projects/fastqc), and sequencing reads were trimmed using the software trimmomatic 0.38 (Bolger et al., 2014). Sequenced reads were aligned to a 2.4 Gb *Lynx canadensis* nuclear reference (mLynCan4_v1.p; GCA_007474595.1; Rhie et al., 2021), following the same workflow as our previous analyses (Bazzicalupo et al., 2022; Lucena‐Perez et al., 2020). Sample location information, as well as sequencing depth and European Nucleotide Archive (ENA) accession numbers for alignment files, can be found in Table S1.

### 2.2 Variant calling and filtering

Variant calling was performed on the genome alignments of all the samples using the GATK v4.1.4.1 (McKenna et al., 2010) HaplotypeCaller command. A first dataset of unfiltered variants consisting of 25.52 million SNPs was then filtered, removing variants found in low mappability and repetitive regions, as well as all INDELs and non‐biallelic variants. Low quality variants were also filtered out following GATK's suggested thresholds of variant quality (i.e., QUAL ≤ 30, QD ≤ 2.0, FS ≥ 60.0, MQ ≤ 40.0), after inspecting the genome‐wide distribution of values of these statistics. Variants that were not represented in at least four samples of each population and with a minimum depth of 3× per sample were filtered out. In order to remove variants in regions where multiple paralogs are collapsed in the reference genome, we removed variants with a read depth exceeding the average read depth plus 1.5 times the standard deviation, calculated using SAMtools depth (Li et al., 2009). The filtered VCF dataset containing the genotype information of all the individuals (whole‐set VCF) consisted of 4,983,054 SNPs. From this whole‐set VCF we then proceeded to exclude some of the populations defined in Lucena‐Perez et al. (2020) and Bazzicalupo et al. (2022) in order to carry out Genome Environment Association (GEA) analyses with confidence (see “Identifying candidate regions of selection” below). We excluded the Balkans, Carpathians, North‐Eastern Poland, and Norway populations, which reduced the sample size of the selection scans to 71, with the remaining 8 populations represented by a minimum of 7 and a maximum of 13 individuals. The excluded populations were characterized by high differentiation, lowest levels of genetic diversity and small recent effective population sizes, caused by intense genetic drift (Bazzicalupo et al., 2022; Lucena‐Perez et al., 2020), a process that may confound the genomic signals of positive selection (Huber et al., 2016; Weigand & Leese, 2018). We finally proceeded to remove all the variants that were below a minimum allele frequency (MAF) of 0.05, yielding a final VCF consisting of 2,100,553 SNPs (final‐set VCF).

### 2.3 Environmental predictors

#### 2.3.1 Extracting environmental data

In order to analyze the association between the genotype and the environment, we extracted environmental data from the geographic area around the coordinates of each of the sequenced individuals using the R package “raster” (Hijmans, 2021). As possible environmental predictors of genetic variation, we used the set of 19 WorldClim bioclimatic variables (Fick & Hijmans, 2017; see Table S2 for the complete list of WorldClim variables used, together with their definition and abbreviation adopted in this work). Additionally, we included two variables related to snow precipitation, the mean number of days with snow cover throughout the year (Mean_snow_days) and the average snow depth during the month of January (Jan_mean_depth). These variables were associated with genetic structure in a previous study (Ratkiewicz et al., 2014) and their values for the years between 1999 and 2018 were extracted from the Northern Hemisphere subset of the Canadian Meteorological Centre operational global daily snow depth analysis (Brown & Brasnett, 2010). Environmental predictor values for each individual were calculated by averaging the values within a circle with a diameter of 100 km around the coordinate location of the individual using the R package “geobuffer” (Ștefan, 2019), a diameter that corresponds to the minimal home range described for the species (Breitenmoser & Breitenmoser‐Würsten, 2008).

#### 2.3.2 Variable selection

In order to select which of the WorldClim environmental predictors had the strongest effect on the genetic variance observed in lynx populations, we implemented a predictive approach based on forward selection using redundancy analysis (RDA), as described by Capblancq and Forester (2021). Briefly, environmental predictors are added to an empty RDA model one by one selecting the one that increases the variance explained by the model the most (in terms of adjusted *r* ^2^ values), until the variance explained by the full model including all the environmental predictors is reached or a permutation‐based significance test does not reach the desired significance threshold. We implemented this using the functions *rda* and *ordiR2step* of the package “vegan” (Oksanen et al., 2020), using a *p*‐value cut‐off of 0.01 and 1000 permutations. Additionally, given their reported importance in determining genetic structure (Ratkiewicz et al., 2014), we decided to add to our final set of environmental predictors the snow‐related variable which would increase the variance explained by the forward‐selected RDA model the most. To reduce collinearity among our environmental predictors, we excluded from the analysis any variable whose variance inflation factor (VIF) exceeded 7 and Pearson's correlation coefficient (*r*) exceeded 0.85 with any other variable, keeping in the analysis the variable with highest adjusted *r* ^2^ contribution to the RDA model.

#### 2.3.3 Variance partitioning

The relative contribution of three different components, geographic location (I), environmental conditions (II) and neutral genetic structure (III), to the overall genetic variation in Eurasian lynx was explored using an approach based on partial RDA (pRDA). The three components were represented by three sets of variables: (I) x and y coordinates for geographic location, (II) the selected environmental predictors for environmental conditions, and (III) the principal components selected using a broken stick model of a genetic PCA run with a set of neutral SNPs. To construct the neutral dataset we extracted the SNPs belonging to intergenic regions (defined as in Lucena‐Perez et al. (2020)) from our whole‐set VCF and excluded any SNP with a minor allele frequency of <0.05, and two samples that did not have any registered geographic location (Table S1). Variance explained by partial models built using one of the three components while controlling for the other two (pure geography, pure climate, and pure structure models) were compared to a model built using the three components together as explanatory variables (full model).

### 2.4 Identifying candidate regions of selection

To identify regions of the genome involved in local adaptation to the environment, we conducted genome‐environment association (GEA) tests. With the aim of improving the true discovery rate, we integrated a multivariate GEA analysis using all of selected environmental predictors at the same time, with multiple GEA analyses considering each individual predictor separately (Forester et al., 2018).

#### 2.4.1 Multivariate approach—redundancy analysis

For the multivariate GEA test we conducted Redundancy Analysis (RDA), a method that extracts and summarizes the variation in a set of response variables that can be explained by a set of explanatory variables, using the SNPs in our final‐set VCF as the response variables and the selected environmental predictors as the explanatory variables. To avoid possible confounding factors introduced by the effects of neutral genetic relationships among populations and based on the observed correlations between genetic structure and geographic location (Figure S1 and Figure S2), we opted to add the first two principal components of the neutral genetic structure as conditioning variables in the RDA model. To avoid collinearity among response variables, our final‐set VCF was pruned using the *indep‐pairwise* option of the software PLINK v.1.9 (Purcell et al., 2007), with a window size of 5 kbp, a step size of one and an *r* ^2^ threshold of 0.7, which resulted in a set of 966,029 non‐correlated SNPs. These were converted to RAW format using the software PLINK v.1.9 (Purcell et al., 2007), and input into R (R Core Team, 2021) using the package “adegenet” (Jombart, 2008). RDA was carried out in R using the package “vegan” (Oksanen et al., 2020). Significance of RDA axes was calculated with an ANOVA like permutation test for Constrained Correspondence Analysis, using the function *anova.cca* and 100 permutations. SNPs with loading values exceeding ±3 standard deviations from the mean on the significant axes were identified as outliers in the selection test and considered as candidates for selection driven by the environmental predictors included in the analysis.

#### 2.4.2 Analysis of individual environmental predictors—BayPass

GEA tests for each of the selected environmental variables were carried out using the software BayPass v2.1 (Gautier, 2015). This software uses Allele Frequency data for different populations and Bayesian Hierarchical Models to generate a distribution of SNPs differentiation coefficients across the genome and correct for underlying genetic differentiation among populations using a scaled population covariance matrix. The Auxiliary Variable Covariate model (AUX) in BayPass implements a binary auxiliary variable δ, whose value indicates if a particular SNP is associated to the tested covariate or not. From the posterior mean of this variable, a Bayes Factor (BF) is derived, with higher values representing higher probability of association between SNP and covariate. The populations for the analysis were defined as in Lucena‐Perez et al. (2020) and Bazzicalupo et al. (2022). To avoid issues with multiple testing and auto‐correlated nearby SNPs, our final‐set VCF was divided into distinct subsets, each including only one of every 50 SNPs, for a total of 50 distinct datasets.

Signals of association with a particular environmental predictor shown by adjacent markers were combined in genomic windows using the R package “GenWin” (Beissinger et al., 2015). GenWin detects inflections in the fitted spline of the underlying summary statistics calculated on a locus‐by‐locus basis (i.e. BF values). These inflection points are then used as window boundaries, allowing us to delimit genomic windows of variable length which contain SNPs responding similarly to each environmental predictor. A new summary statistic is calculated for each window (Wstat), whose value will depend on the mean BF of SNPs within the window, weighted by the mean and standard deviation of the entire dataset and the number of SNPs inside of the window. The function *splineAnalyze* was run to calculate window boundaries and Wstat along each chromosome, and to correct for the whole‐genome mean and variance of BF values, with a resolution of 10 kbp in spline calculation and generalized cross validation. Windows with a Wstat value exceeding the 99th percentile of the overall distribution were considered as outliers and thus candidate selected regions.

#### 2.4.3 Generating a final set of candidate windows and SNPs

We intersected the results of the analyses of the individual environmental predictors with the results of the multivariate analysis and kept as candidate windows for local adaptation only the outlier windows from the former which also contained at least one outlier SNP from the latter. From each of the candidate windows, we then proceeded to extract the SNP showing the highest association with the environment, based on absolute load values of the RDA axis for which they were found as outliers. This gave us a final set of 394 candidate windows and a corresponding set of 394 candidate SNPs to use in further analyses.

To conduct comparative analyses between adaptive and neutral genetic variation we also extracted a set of neutral SNPs. To construct the neutral dataset we extracted the SNPs belonging to intergenic regions (defined as in Lucena‐Perez et al. (2020)) from our whole‐set VCF, proceeded to exclude any SNP that was recovered as an outlier in any of our tests and any SNP with a minor allele frequency of <0.05. From this set we extracted a random subset of 1500 SNPs, in order to have a number of SNPs in our neutral dataset similar to that in our candidate dataset.

### 2.5 Functional enrichment of candidate genes

To explore what biological functions were associated to the genes with candidate SNPs, we used the PANTHER classification system (Mi et al., 2021), which classifies genes based on their Gene Ontology (GO) term annotation, such as molecular or biological function, and their relationships with other genes, through the classification of gene families and pathways (Ashburner et al., 2000; The Gene Ontology Consortium et al., 2021). The list of candidate genes involved in local adaptation was extracted by crossing our candidate windows, extended to include 20 kbp up‐ and down‐stream adjacent sequences, with the NCBI *L. canadensis* reference genome annotation (Rhie et al., 2021). Statistical over‐ and under‐representation tests for each Biological Process GO term were carried out through the PANTHER platform, by comparing the expected number of occurrences of a given GO term based on the total set of genes in the reference genome with that observed in the candidate gene list. Only significant (*p*‐value < 0.05) over‐ and under‐representations are reported.

### 2.6 Adaptive population structure

To understand the effects that local adaptation has on the genetic structure of the different Eurasian lynx populations, two PCAs were run using different sets of SNPs as input data, one for the potentially adaptive SNPs and one for the neutral SNPs. Data preparation and PCA analysis were carried out using the software PLINK v1.9. The results were represented in R using the package “ggplot2” (Wickham, 2009). To understand how local adaptation changed the relationships among individuals from different populations compared to their background genetic differentiation, we calculated pairwise Euclidean distances between all the individuals based on the neutral and the adaptive datasets and compared them. To allow the distances to be comparable between datasets, we used a random subset of 394 neutral SNPs to have the same number in the two sets.

### 2.7 Analysing spatial gradients of adaptive genetic differentiation

Turnovers in neutral and adaptive genetic composition across the distributional range of the Eurasian lynx as a response of both environmental conditions and geographic location were analyzed through General Dissimilarity Modelling (GDM). GDM is a distance based method that tries to estimate how a response variable (i.e. adaptive and neutral genetic variation) changes between sites because of the effect of a set of explanatory variables (i.e. geographic location and environmental predictors), by identifying which environmental gradient explains most of the biological variation, and determining where along the gradient the turnover is slower or faster (Fitzpatrick & Keller, 2015). Two Euclidean distance matrices between our samples were built, one from our set of 394 candidate SNPs and one from our neutral SNPs, and then used as the response matrices. Neutral and candidate variant data were extracted for all of our samples, except for the two samples that did not have any registered geographic location (Table S1). The analysis was run in R using the package “gdm” (Fitzpatrick et al., 2021) using the full set of 21 environmental predictors together with the geographic coordinates of each sample as explanatory variables. The relative importance of each geographic and environmental predictor was estimated as the rescaled maximum value of the model's fitted I‐Splines, which are proportional to variable importance (Fitzpatrick & Keller, 2015). Environmental predictors raster layers were then transformed according to the turnover function estimated by the GDM using the function *gdm.transform*. To summarize and visualize GDM results, the principal components of variation were extracted with PCA of the transformed raster layers. The function *plotRGB* was then used to generate Red‐Green‐Blue plots based on the first three principal components, allowing the visualization of predicted turnover in genetic composition in each dataset. To analyze how the gradients of genetic compositions compared between neutral and candidate SNPs, we performed a Procrustes superimposition on the resulting PCA ordinations and mapped its residuals, as a measure of local difference in predicted turnover in genetic compositions between datasets, as described in Fitzpatrick and Keller (2015).

## 3 RESULTS

### 3.1 Variable selection and variance partitioning

A total of five environmental predictors were selected by the forward model building approach (Table S3), Mean Temperature of Driest Quarter (T_dry_quart), Temperature Mean Diurnal Range (T_range_day), Precipitation Seasonality (P_seasonality), Precipitation of Wettest Quarter (P_wet_quart), and Precipitation of Wettest Month (P_wet_month). From this set we opted to exclude P_wet_month for its high VIF score (59.4) and high correlation with P_wet_quart (*r* = 0.98), and lowest contribution to the variance explained by the RDA model (Figure S2 and Table S3). As the environmental predictor related to snow, we added the mean number of days with snow cover throughout the year (Mean_snow_days) to the four variables selected in the previous process, because of its slightly higher contribution to the RDA model (*r* ^2^ = 0.166) when compared to the average snow depth during the month of January (*r* ^2^ = 0.165).

The first two principal components of the neutral PCA were selected by the broken stick model to represent neutral genetic structure in the pRDAs (Figure S1). The analysis of variance partitioning showed that the full model, including all three components of geographic location, environmental conditions, and neutral genetic structure, explained 26% of the total variance (Table S4). All the partial models were significant, with the pure climate one explaining the highest proportion of variance (33% of the explainable and 9% of the total variance), indicating a net significant influence of the environment on the genetic composition of different Eurasian lynx populations. The pure genetic structure and pure geography partial models explained less variance but still a substantial amount, with 22% of explainable variance and 6% of total variance for the genetic structure model and 17% of explainable variance, 4% of total variance for the geography model, indicating a similarly strong effect of isolation by distance, genetic isolation and drift on the genetic variation observed. Based on the observation of this strong effect, and the high correlation between geography and the first two principal components of the neutral genetic structure (Figure S2), the RDA models that were run for the multivariate GEA analyses included a correction for the first two principal components of the neutral genetic structure as proxies of this neutral and geographic differentiation. The rest of the variance (29% of explainable, 7% of total) could not be explained by any one of the three sets of predictors uniquely, most probably because of the collinear relationships among them (Figure S2).

### 3.2 Genomic scans of selection

A total of 2,100,553 autosomal SNPs passed our stringent filtering criteria and were used as input for genomic scans of selection. This set had a total genotype calling rate of 99.76%, with per‐sample genotype missing rates not exceeding 0.1%. These were pruned into a non‐correlated set of 966,029 SNPs with no missing data for multivariate RDA and into 50 independent datasets for the univariate BayPass analyses.

The multivariate RDA (*r* ^2^ = 0.11) resulted in two significant RDA axes, which together accounted for over 60% of the constrained variance in the model (Figure S3). A total of 6244 unique loci were identified as outliers by having loadings ±3 standard deviations from the mean, of which only 7 were found on RDA1, while the remaining 6236 were found on RDA2. Figure S4 highlights outlier SNPs on the two axes of ordination, showing which environmental predictor they more strongly correlate with.

The separate BayPass analyses identified around 1300 outlier windows for each of the environmental predictors, widely distributed throughout the genome (Figure S5 and Table S5). Mean window size was around 20 kbp (range: 10,000–90,000), not varying much for the different predictor variables (Table S5). Outlier windows from the separate BayPass tests on each predictor variable were then intersected with outlier SNPs from the multivariate RDA analysis (Figure S5). The total number of candidate windows intersecting with at least one multivariate outlier SNP was 108 for T_range_day, 96 for T_dry_quart, 54 for P_seasonality, 68 for P_wet_quart, and 279 for Mean_snow_days (Tables S5 and S6). By joining all outlier windows from the five environmental predictors, we obtained a total of 394 unique candidate windows, from which we extracted our 394 candidate SNPs dataset, each representing one window.

### 3.3 Functional enrichment of candidate genes—panther overrepresentation test

A total of 782 unique genes overlapped the candidate windows, of which 658 were mapped by PANTHER to a unique Biological Process GO category. A total of 37 distinct GO terms were found to be over‐represented in our candidate gene set (FDR < 0.05; Table 1). Of these, the highest fold enrichment (>2) was found for GO terms associated with protein O‐linked glycosylation (GO:0006493), behavior (GO:0007626, GO:0007610), synaptic regulation and organization (GO:0050808, GO:0050804, GO:0099177), and nervous system development (GO:0007420, GO:0060322, GO:0007417, GO:0007399).

**TABLE 1:** Table summarizing the results of PANTHER over‐representation analysis.

GO biological process complete	Reference	Input	Expected	Fold enrichment	Raw *p*‐value	FDR
Protein O‐linked glycosylation (GO:0006493)	60	7	0.95	7.35	<0.0001	<0.05
Locomotory behavior (GO:0007626)	150	11	2.38	4.62	<0.0001	<0.05
Synapse organization (GO:0050808)	209	13	3.32	3.92	<0.0001	<0.05
Modulation of chemical synaptic transmission (GO:0050804)	290	18	4.60	3.91	<0.0001	<0.005
Regulation of trans‐synaptic signaling (GO:0099177)	290	18	4.60	3.91	<0.0001	<0.005
Behavior (GO:0007610)	416	20	6.60	3.03	<0.0001	<0.05
Brain development (GO:0007420)	484	21	7.68	2.73	<0.0001	<0.05
Head development (GO:0060322)	527	22	8.37	2.63	<0.0001	<0.05
Central nervous system development (GO:0007417)	672	28	10.67	2.62	<0.0001	<0.005
Nervous system development (GO:0007399)	1455	50	23.10	2.16	<0.0001	<0.005
System development (GO:0048731)	2713	81	43.07	1.88	<0.0001	<0.0005
Multicellular organism development (GO:0007275)	3054	86	48.49	1.77	<0.0001	<0.0005
Animal organ development (GO:0048513)	2321	65	36.85	1.76	<0.0001	<0.005
Regulation of cell communication (GO:0010646)	2522	68	40.04	1.70	<0.0001	<0.05
Regulation of signaling (GO:0023051)	2535	68	40.25	1.69	<0.0001	<0.05
Regulation of biological quality (GO:0065008)	2806	74	44.55	1.66	<0.0001	<0.05
Anatomical structure development (GO:0048856)	3707	95	58.85	1.61	<0.0001	<0.005
Developmental process (GO:0032502)	4015	101	63.74	1.58	<0.0001	<0.005
Localization (GO:0051179)	3745	89	59.46	1.50	<0.0001	<0.05
Multicellular organismal process (GO:0032501)	5071	119	80.51	1.48	<0.0001	<0.005
Cellular component organization (GO:0016043)	4374	102	69.44	1.47	<0.0001	<0.05
Cellular process (GO:0009987)	13,432	247	213.25	1.16	<0.0001	<0.05
Biological_process (GO:0008150)	16,132	282	256.12	1.10	<0.0001	<0.05

*Note*: Gene Ontology codes with FDR scores <0.5 and their definitions are given for the set of genes found to be associated to local adaptation, sorted by descending fold enrichment.

### 3.4 Adaptive population structure

**FIGURE 2:**
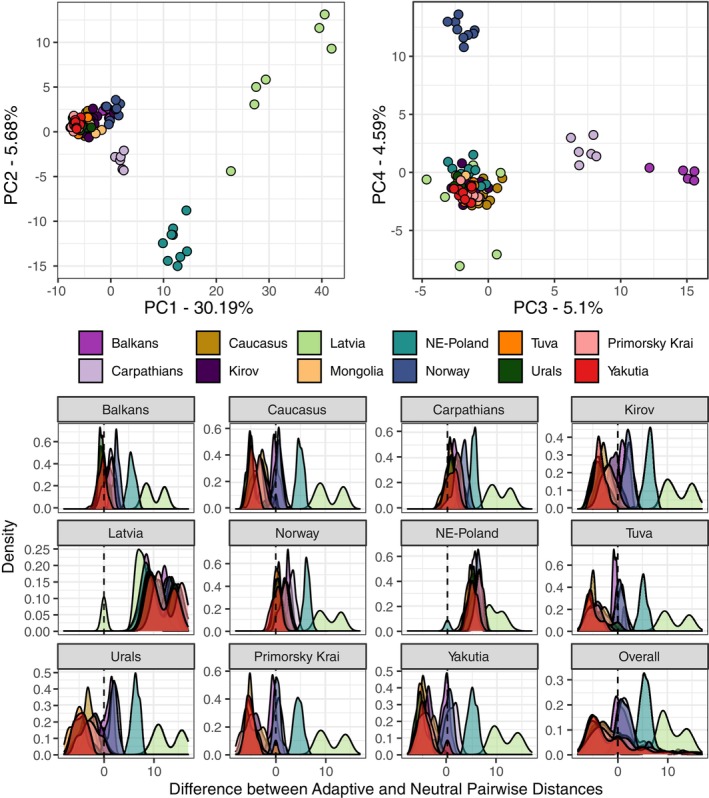
Adaptive genetic structure as recovered by PCA (top) and population frequency distributions of differences in pairwise Euclidean distances between neutral and adaptive loci (bottom). In the PCA biplots relationships between samples, color coded by population, are represented for the first two principal components (top left) and the third and fourth principal components (top right). (bottom). A frequency distribution graph of the difference between adaptive and neutral distances between the members of the population and the individuals of each of the other populations (color‐coded). A vertical dashed line at the zero separates pairs of populations that are adaptively similar but neutrally divergent, whose differential distances are zero or negative, and adaptively divergent pairs, whose differential distances are positive.

Genetic structure recovered from a subset of neutral loci (Figure S6) differed substantially from that reconstructed from loci potentially involved in local adaptation (Figure 2). All of the samples belonging to the populations of the Eastern neutral clade (Mongolia, Tuva, Primorsky krai, Yakutia), together with part of the samples from the Western clade (Kirov, Urals) and the Caucasian population, form a relatively homogeneously adapted group, with similar values of the first four principal components of the adaptive PCA (Figure 2). Furthermore, these populations are consistently more similar to each other when looking at the adaptive loci compared to neutral loci, as reflected by negative differences between Euclidian distances between the two sets (Figure 2). Samples from the Balkans, Carpathians and Norway populations retain similar differentiation to this homogeneously adapted group in the adaptive and neutral datasets, with differences among Euclidian distances around zero between the two, and they differentiate from the rest of populations in either the third (Balkans and Carpathians) or the fourth (Norway) principal components of the adaptive PCA. The Latvia population, which was recovered relatively close to the other Eurasian lynx populations using the neutral genetic data, appears separated from the rest of populations in the first and partially in the second principal components of the adaptive PCA, and is consistently the most distant to all other populations in the adaptive space. The population of NE‐Poland also differentiated itself in the first two principal components of the adaptive PCA, occupying a middle position between Latvia and the rest of populations in the first principal component, as well as occupying the negative extreme of the second principal component. It is also, following Latvia, the second most adaptively distant population to the rest of Eurasian lynx populations. Overall, as the first principal component explained around 30% of the total variation in the adaptive genetic dataset, which represented more than five times the amount explained by the second principal component (<6%), Latvia and NE‐Poland are the most and second most adaptively divergent populations, respectively. Other than these two, most populations (Caucasus, Kirov, Mongolia, Primorsky Krai, Tuva, Urals, Yakutia) belong to an adaptively homogeneous group, and the three remaining populations (Balkans, Carpathians, Norway) are slightly differentiated from each other and from the homogeneous group, but to a much smaller degree than NE‐Poland and Latvia.

### 3.5 Spatial gradients of adaptive genetic differentiation—GDM

**FIGURE 3:**
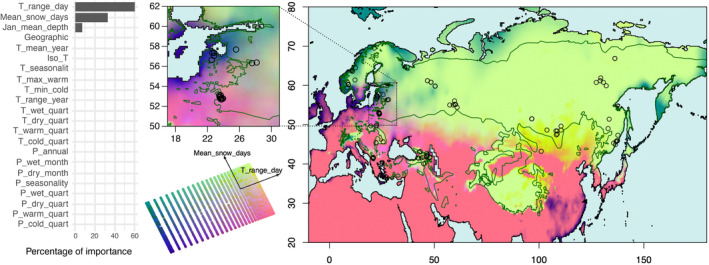
Predicted turnover in genetic composition as calculated by GDM on adaptive genetic markers. Locations with similar colors are expected to have similar adaptive genetic composition, based on the effects of the different environmental predictors. The contribution of different variables to genetic composition is indicated in the associated biplot, with vectors pointing to the direction of the contributing variable and indicating the magnitude of its contribution. The bar plot indicates the relative importance of each environmental variable to the overall GDM model calculated as the rescaled maximum value of the model's fitted I‐Splines. Circles represent sampled individuals, and a green line delimits Eurasian lynx current distributional range (Breitenmoser et al., 2015). A map zoomed on the range occupied by the adaptively divergent Latvia and NE‐Poland populations is presented to appreciate their distinct expected genetic compositions.

Results from the GDM analysis allow us to visualize the expected variation in genetic composition in geographic space (turnover) as a response to geographic location and different environmental predictors (Figure 3 and Figure S7). The percentage of null deviance explained by the fitted GDM model was 57.25% for the neutral dataset and 62.14% for the adaptive dataset. Biplots of the first two principal components with labelled vectors are also included in Figure 3 and Figure S7, giving an idea of the magnitude and direction of the most correlated environmental predictors. Relatively homogeneous turnover is observed for neutral genetic composition (Figure S7). Overall, changes in neutral genetic compositions are predicted to be mainly a consequence of geographic location (Figure S7), affected by both latitude and longitude (biplot in Figure S7 and Table S7). Other environmental predictors have much lower relative importance, with the highest non‐geographic predictors being isothermality (Iso_T), with less than half the relative importance of geography (Figure S7).

In sharp contrast to neutral variation, the turnover of adaptive genetic composition is greatly influenced by two environmental predictors, T_range_day and Mean_snow_days, with 60.35% and 32.77% of the overall importance respectively (Figure 3 and Table S8). Throughout most of the Northern part of the species range, adaptive genetic composition is determined by the effect of high values of Mean_snow_days, with predicted composition shifts between areas affected by lower or higher values of T_range_day (range of darker to lighter green in Figure 3, see biplot). Most of the populations sampled in this study fall within these regions of high values of both these variables, including all the Asian populations (Tuva, Mongolia, Yakutia, Primorsky Krai), the populations from Western Russia (Kirov, Urals) and Scandinavia (Norway). Patches of similar adaptive compositions are predicted to be found in highland regions of the Southern part of the range. These are the mountainous ranges of Europe (occupied by the Carpathian and Balkan populations), as well as the Caucasian and Himalayan mountains. The rest of the Southern range is characterized by either lower Mean_snow_days but high T_range_day, as in the lowland areas of the European range (where we sampled the NE‐Poland population and part of the Latvia population), Anatolia, and Iran (pink areas in Figure 3, see biplot) or lower values of both, as in the Latvian Courland Peninsula (where the rest of the Latvia population is sampled) and the southern tip of Scandinavia (purple and blue areas in Figure 3, see biplot). The differences in turnover patterns between the neutral and adaptive datasets are highlighted by the geographical representation of Procrustes superimposition residuals (Figure S8). Higher residual values are observed as we move along the longitudinal range, as the main difference is represented by the effect of geography, which strongly influences neutral genetic composition, but does not affect the adaptive one.

## 4 DISCUSSION

In this study, we examined range‐wide patterns of adaptive differentiation driven by environmental conditions across the distributional range of the Eurasian lynx. Loci potentially involved in local adaptation were identified through their association with environmental predictors. We assessed how local adaptation in Eurasian lynx populations affects global genetic structure of this large carnivore species by describing the way neutral and adaptive genetic differentiation patterns differ, and by estimating both the magnitude and the direction of these changes. We also described how genetic composition is expected to change throughout the species' range in response to geographic distance and environmental factors. By analyzing the changes in adaptive genetic compositions across the distributional range of the species we discovered areas of expected strong adaptive divergence driven by specific environmental conditions. We also identified the biological processes that were the main targets of this adaptive differentiation as those overrepresented among the genes with variation associated to environmental variables.

### 4.1 Evidence of local adaptation

The genetic structure reconstructed with candidate adaptive loci was substantially different to that reconstructed with neutral ones. The three main clades recovered in the neutral genome, that divide Eurasian lynx populations in three main lineages (Bazzicalupo et al., 2022; Lucena‐Perez et al., 2020) are not observed when looking at the areas of the genome identified as responding adaptively to environmental conditions. From the adaptive PCA a highly homogeneous group is instead recovered, which includes representatives from three distinct neutral clades, the Caucasus, the Western lynx clade (Kirov, Urals) and the Eastern lynx clade (Mongolia, Primorsky Krai, Tuva, Yakutia), with adaptive genetic distances being lower than neutral genetic distances in all pairwise comparisons. A similar pattern is also observed when mapping the turnover in expected genetic composition in response to environmental conditions. Across the north‐western, central, and eastern part of the distribution, areas occupied by lynxes of this adaptively homogenous group, genetic composition is modelled to be mostly homogeneous and largely driven by a high mean number of days with snow cover (Mean_snow_days) and high mean diurnal temperature ranges (T_range_day). Given the low adaptive distance among the populations and the similar environmental pressures they seem to face, it appears as the neutral differentiation observed among these populations is consistent with a scenario of pure neutral evolution driven by historical isolation and limitations in gene flow caused by distance, as previously discussed (Bazzicalupo et al., 2022; Lucena‐Perez et al., 2020). On the other hand, the variants involved in the adaptation to local environmental conditions maintain more similar allelic frequencies among neutrally differentiated populations and may be thus more resistant to genetic drift. The lack of signals of differential adaptation within this adaptively homogeneous populations contrasts with the extensive variation in phenotype (e.g. pelage pattern and size) and in habitat and prey across the species' wide distributional range (Breitenmoser & Breitenmoser‐Würsten, 2008; Darul et al., 2022; Kitchener et al., 2017). This suggests that either the observed phenotypic variation is evolving as neutral, or that it is a plastic response to the environment. In fact, pelage pattern variation was found to be poorly associated to local habitat and most closely related to least‐cost distances from inferred glacial refugia, suggesting that the current patterns of pelage variation are more of a legacy of past phylogeographic history since the last glacial maximum than the outcome of local adaptation to the environment (Darul et al., 2022). On the other hand, epigenetic differentiation was found to be responsible for the variation in size between Alaskan and insular Newfoundland populations of the closely related Canada lynx, in a background of neutral homogeneity (Meröndun et al., 2019). This suggests that even though we are not observing adaptive divergences among Eurasian lynx in the north‐west, central, and eastern parts of the distribution, environmental conditions could still be potentially inducing adaptive plastic responses in phenotypic traits through epigenetic changes. Additionally, environmental variables not included in this study, such as soil type or land cover, could also be driving local adaptations within this homogeneous group that we have not been able to detect in this study. Overall, it seems that ecological flexibility might have allowed this homogeneous group to colonize widespread areas with diverse climatic and ecological conditions. It will be interesting to address whether that is also the case for other felids, such as jaguars (Eizirik et al., 2001) and pumas (Culver, 2000), widespread species which may also base their evolutionary success as apex predators on phenotypic plasticity rather than natural selection on genomic variants.

Most of the divergence observed in adaptive loci is represented by the differentiation of the samples belonging to the westernmost region of the Baltic population of Eurasian lynx, represented in our study by the Latvia and NE‐Poland populations. Samples from both populations are differentiated in the adaptive PCA and show higher adaptive than neutral distances from the rest of samples. Genetic composition in the regions occupied by these populations is again mostly driven by two environmental predictors, the mean number of days with snow cover throughout the year (Mean_snow_days) and the ranges in mean diurnal temperature (T_range_day). Low values of both variables are expected to determine the genetic composition of lynx inhabiting the Latvian Courland Peninsula, while higher Mean_snow_days characterize the rest of the Latvia population and higher T_range_day influences the genetic composition of NE‐Poland. The high adaptive differentiation observed in these populations and the distinctive environmental conditions they are subjected to suggest an ongoing process of adaptive differentiation in the regions occupied by these populations. This contrasts with what is observed when looking at the neutral genome, where individuals from Latvia and NE‐Poland are relatively closer to other members of what is known as the European lowland or Baltic lynx populations, which also includes members of the Western‐Russia populations of Kirov and Urals (Lucena‐Perez et al., 2020; Schmidt et al., 2021). Some evidence of genetic structuring between the westernmost Baltic lynx individuals and the Scandinavian and West‐Russian was already detected when reconstructing their mitochondrial phylogenomic relationships, which revealed the presence of at least two distinct haplogroups (Lucena‐Perez et al., 2020; Mengüllüoğlu et al., 2021), one of which is in fact more related to other Eurasian lynx from the Asian part of the distributional range. It could be possible that adaptive divergence to different environmental conditions occurred during the isolation of these more anciently divergent mitochondrial clades in separate refugia with different environments. Given that the adaptive genetic composition of the previously inhabited lowland areas of the European landscape is predicted to be similar to that of these adaptively divergent populations, these adaptations may have been widespread across Europe in the past. In such case, we would now be observing the remnants of a differentially adapted lowland lynx following the extensive habitat reduction and fragmentation that occurred in the area. Gene‐flow between these adaptively diverging populations is however still ongoing, as demonstrated by their sharing of mitochondrial haplotypes with other western lynx and their relative homogeneity in the neutral genome (Lucena‐Perez et al., 2020; Mengüllüoğlu et al., 2021).

Weaker signals of differentiation in the adaptive PCA are observed for individuals of the Balkans, Carpathians, and Norway populations, whose adaptive distances to members of other populations than their own do not differ from the corresponding neutral distances or are only slightly higher. These populations occupy regions with environmental conditions that more closely resemble the ones observed in the northern‐most part of the distribution, characterized by high Mean_snow_days and T_range_day, as the consequence of either their altitudinal position, in the mountainous ranges of the Carpathians and the Balkans, or their northward latitudinal position, in the case of Norway. As their neutral and adaptive distances to the rest of populations are of similar magnitude, and they do not appear to occupy environments with unique climatic conditions, their overall genomic differentiation is most parsimoniously interpreted as the consequence of genetic drift augmented by isolation and low effective population sizes. It must be noted, however, that these populations were excluded from our search of adaptive variation specifically because of intense recent genetic drift, so we may have missed potential variants underlying adaptations exclusive to these populations.

### 4.2 Drivers of local adaptation and genes involved

Variance partitioning analysis of the RDA model, although limited by the number of observations we have compared to the number of variables considered, allowed us to disentangle the effects of demographic history, geographic location, and environmental conditions on genetic variation in Eurasian lynx, and revealed that part of the genetic variation among individuals could be attributed to local climatic conditions. Gradients in annual mean diurnal ranges in temperature (T_range_day) and the mean number of days with snow cover throughout the year (Mean_snow_days) appear to have the strongest effects on adaptive genetic variation of Eurasian lynx populations, indicating that these environmental variables – or others closely correlated with these (see below) – might be important factors for the biology and evolution of this species.

Apart from its previously reported correlations with skull morphology and diet (Yom‐Tov et al., 2011), snow precipitation was also already suggested as a possible predictor of genetic differentiation in the western range of the Eurasian lynx, based on both mtDNA and microsatellite markers (Ratkiewicz et al., 2014; Schmidt et al., 2011). It was suggested that familiarity and adaptation to local snow conditions would increase efficiency in prey capture and discourage dispersal to other climatic regions (Nilsen et al., 2009), similarly to how snow conditions are predicted to affect population differentiation and dynamics in Canada lynx (Stenseth, Ehrich, et al., 2004; Stenseth, Shabbar, et al., 2004). It is hard to predict the selective pressures that diurnal ranges in temperature generate on lynx populations.

The identification of Mean_snow_days and T_range_day as the most important variables in shaping adaptive genetic composition should be taken with a grain of salt, as it might be the result of an indirect correlation. Other climatic or ecological variables correlated to these two might be the actual causal factors, and the effect we observe here could be a byproduct of their correlation. In particular, local adaptations in response to T_range_day might be associated with specific mechanisms that determine how Eurasian lynx directly cope with temperature fluctuations. On the other hand, T_range_day might represent a proxy of ecological environments, as diurnal ranges in temperature have been linked to forest structure heterogeneity (Ehbrecht et al., 2019), which ultimately affects the ecological community composition of an area (Levine & HilleRisLambers, 2009). Indeed, a significant effect of the habitat structure, mediated by stalking cover availability, on habitat suitability and on the distribution of Eurasian lynx was recently reported in central Europe (Schmidt et al., 2023). Specialization on the hunting of particular prey, mainly larger ungulates, is being observed for lynx inhabiting the Baltic region (Valdmann et al., 2005), which are here recovered as the most adaptively divergent. Local climatic conditions might be facilitating the acquisition of these larger prey, allowing locally adapted lynx to thrive, and even colonize new territories.

With the data at hand, precise descriptions of the mechanisms by which the genes involved in local adaptation directly affect the fitness of Eurasian lynx cannot be made. Our results suggest that four main categories of biological functions are carried out by the genes involved in local adaptation to the environment: metabolic processing of proteins and sugars by O‐glycosylation, locomotory and general behavior, synaptic regulation and organization, and nervous system development. O‐linked glycosylation is a type of protein glycosylation that adds sugar molecules to specific serine or threonine residues on proteins. This modification can have a very wide range of effects on a number of different biological processes, by influencing protein conformation and expression, by regulating processes mediated by the organisms molecular interaction events, such as fertilization or immune responses, and by modulating the activity of signaling molecules such as hormones (Hounsell et al., 1996; Van den Steen et al., 1998). Strong adaptive pressures on behavior and neural regulation, organization and development might be common among hypercarnivores, as enrichment in genes with similar functions has also been observed when analyzing other carnivores' evolution (Mittal et al., 2019; Scholl et al., 2021). Broadly speaking, local adaptation might be affecting the way lynx are experiencing, navigating, and regulating their physiology in the different ecological conditions imposed by snow, temperature, and other possibly associated ecological variation.

### 4.3 Implications for conservation and taxonomy

The neutral variation observed in the Eurasian lynx and the adaptive variation described in this study are not similar in the way they differentiate among populations and subspecific clades. The divergent groups corresponding to the subspecies of *L. l. lynx* in the west, *L. l. wrangeli* in the east, and *L. l. dinniki* in the Caucasian mountains, revealed by the neutral genome (Bazzicalupo et al., 2022; Lucena‐Perez et al., 2020), are not observed when looking at adaptive variation. Across all of the sampled range, except for the westernmost part of the distribution, these three divergent lineages appear as adaptively homogeneous. Their divergence is therefore most probably a consequence of the varying interaction of genetic drift and gene flow across space and time, it is strongly correlated with geographic location and with isolation in separate glacial refugia, and does not appear to be driven by adaptive processes. As for the remaining subspecies we have sampled, *L. l. balcanicus* from the Balkans and *L. l. carpathicus* from the Carpathian Mountains, we detect some differentiation at adaptive variants, which occurs however along less important axis of adaptive differentiation and is much weaker than that observed with neutral variants. It is thus likely that genetic drift might have affected allele frequencies in these otherwise selected variants (Bazzicalupo et al., 2022; Lucena‐Perez et al., 2020).

The strongest signals of adaptive divergence driven by the environment were observed in the populations in Latvia and NE‐Poland, which seem to be locally adapted to different snow conditions and diurnal temperature fluctuations, experiencing a relatively mild climate when compared, for example, to northern Scandinavia or the mountainous ridges of the Carpathians and the Caucasus. However, without additional support from phenotypic and ecological data, we advise against using this evidence to classify Baltic populations as a novel subspecies separate from *L. l. lynx*.

Apart from taxonomic inferences, understanding how adaptive variation shapes the relationships between different populations of Eurasian lynx can assist the identification of evolutionary significant units (ESUs; Funk et al., 2012; Ryder, 1986) and ecotypes (Stronen et al., 2022), with the aim of guiding conservation management decisions (Teixeira & Huber, 2021). Our results suggest that differences in local environmental conditions, especially linked to snow precipitation and daily temperature fluctuations, should be an important factor to consider when delimiting Eurasian lynx ESUs. Within the distributional range of the *L. l. lynx* subspecies, we were able to identify at least two possibly distinct adaptive units. One of them is adapted to harsher conditions of high snow and temperature fluctuations, which is widely distributed in areas of Western‐Russia and Scandinavia, and the other is adapted to the milder climatic conditions of European lowlands. Conservation and restoration programs might want to take these two adaptive units into consideration when selecting potential sources for reintroduction or reinforcement. In particular, reintroductions in western Europe, currently based mostly on Carpathian lynx (Mueller et al., 2022), might consider the Baltic population as an additional and valuable source. Indeed, the reintroduced lynx population in western Poland is assumed to be founded by individuals that, based on their genotypes, correspond to the Baltic population (Tracz et al., 2021). A more challenging case is represented by the Balkan lynx, *L. l. balcanicus*, whose adaptive differentiation observed in this study is hard to disentangle from the neutral divergence caused by intense genetic drift and prolonged low effective population sizes. Our previous assessment of the Carpathian population as representing the best source of individuals for genetic rescue of this highly endangered population, based on its closest relationship in neutral autosomal variation (Bazzicalupo et al., 2022) and also supported by their ecological similarity (Melovski et al., 2022), could be additionally confirmed by their relative overall adaptive similarity and by their similar environment defined by the most relevant environmental variables.

Our results are also relevant for evaluating the long‐term effects of climate change on the Eurasian lynx distribution. The two environmental variables identified as drivers of adaptive divergence are likely to alter their geographical distribution in the near future due to climate change, so associated variants may also expand and contribute to species resilience and persistence. In other species, the occurrence of adaptive responses to environmental pressures has been shown to reduce range loss projections under future climatic conditions (Razgour et al., 2019). It is thus critically important to conserve the range of adaptive variation within species by protecting populations harboring locally adapted variants if we aim to assure their long‐term viability.

## CONFLICT OF INTEREST STATEMENT

The authors declare no conflicts of interest.

## Supporting information


Appendix S1.

## Data Availability

Raw sequence data is available in the European nucleotide archive (ENA) under the study primary accession number: PRJEB48088. Custom scripts ran during data analysis are available at https://github.com/Enricobazzi/Local_adaptation_Eurasian_lynx.
