# Supplementary material for: Genome‐environment association analyses reveal geographically restricted adaptive divergence across the range of the widespread Eurasian carnivore Lynx lynx (Linnaeus, 1758)
Source: Evol Appl. 2023 Oct 9;16(11):1773–88. doi: 10.1111/eva.13570 (PMC10681490; doi:10.1111/eva.13570)
Supplement: Supplementary file 1 — Appendix S1. [file EVA-16-1773-s001.docx]

**Genome-environment association analyses reveal geographically restricted adaptive divergence across the range of the widespread Eurasian carnivore *Lynx lynx*(Linnaeus, 1758)**

Running Title: Local adaptation in Eurasian lynx

## SUPPLEMENTARY MATERIAL

### Supplementary Tables

**Table S1.** Samples used in this study. The table includes the population, country of origin, the spatial coordinates, sex (f, female; m, male), coverage depth of nuclear data (average reads per site).

| **Sample** | **Population** | **Subspecies** | **Country** | **X coord** | **Y coord** | **Sex** | **Coverage depth** | **Used in GEA** |
| --- | --- | --- | --- | --- | --- | --- | --- | --- |
| c_ll_ba_0224 | Balkans | *L. l. balcanicus* | Macedonia | 20.77 | 41.48 | f | 31.7 | no |
| c_ll_ba_0226 | Balkans | *L. l. balcanicus* | Macedonia | 20.96 | 41.50 | f | 14.71 | no |
| c_ll_ba_0227 | Balkans | *L. l. balcanicus* | Macedonia | 20.82 | 41.38 | f | 16.48 | no |
| c_ll_ba_0228 | Balkans | *L. l. balcanicus* | Macedonia | 20.94 | 41.63 | m | 27.24 | no |
| c_ll_ba_0229 | Balkans | *L. l. balcanicus* | Macedonia | 20.79 | 41.48 | m | 21.33 | no |
| c_ll_ba_0230 | Balkans | *L. l. balcanicus* | Macedonia | 20.79 | 41.48 | m | 19.91 | no |
| c_ll_ba_0233 | Balkans | *L. l. balcanicus* | Albania | na | na | m | 21.5 | no |
| c_ll_ca_0240 | Caucasus | *L. l. dinniki* | Georgia | 42.49 | 41.46 | m | 34.6 | yes |
| c_ll_ca_0241 | Caucasus | *L. l. dinniki* | Georgia | 46.38 | 41.87 | f | 21.96 | yes |
| c_ll_ca_0242 | Caucasus | *L. l. dinniki* | Armenia | 44.93 | 40.03 | f | 22.11 | yes |
| c_ll_ca_0243 | Caucasus | *L. l. dinniki* | Russia | 47.84 | 41.57 | m | 27.3 | yes |
| c_ll_ca_0244 | Caucasus | *L. l. dinniki* | Russia | 47.89 | 41.73 | f | 18.16 | yes |
| c_ll_ca_0245 | Caucasus | *L. l. dinniki* | Russia | 46.67 | 42.39 | f | 8.49 | yes |
| c_ll_ca_0247 | Caucasus | *L. l. dinniki* | Georgia | 44.35 | 41.69 | m | 13.07 | yes |
| c_ll_ca_0248 | Caucasus | *L. l. dinniki* | Georgia | 42.81 | 43.04 | f | 11.3 | yes |
| c_ll_ca_0252 | Caucasus | *L. l. dinniki* | Russia | 43.25 | 43.20 | f | 16.91 | yes |
| c_ll_ca_0254 | Caucasus | *L. l. dinniki* | Georgia | 42.06 | 41.57 | m | 14.45 | yes |
| c_ll_ca_0259 | Caucasus | *L. l. dinniki* | Russia | 46.39 | 42.69 | m | 12.3 | yes |
| c_ll_ca_0260 | Caucasus | *L. l. dinniki* | Russia | 46.45 | 42.10 | m | 16.72 | yes |
| c_ll_cr_0205 | Carpathians | *L. l. carpathicus* | Poland | 19.76 | 49.67 | f | 8.17 | no |
| c_ll_cr_0206 | Carpathians | *L. l. carpathicus* | Romania | 25.60 | 45.64 | m | 7.86 | no |
| c_ll_cr_0207 | Carpathians | *L. l. carpathicus* | Romania | 22.20 | 45.40 | f | 8.95 | no |
| c_ll_cr_0208 | Carpathians | *L. l. carpathicus* | Poland | 22.30 | 49.75 | f | 7.82 | no |
| c_ll_cr_0209 | Carpathians | *L. l. carpathicus* | Romania | 22.20 | 45.40 | f | 8.84 | no |
| c_ll_cr_0211 | Carpathians | *L. l. carpathicus* | Romania | na | na | m | 24.02 | no |
| c_ll_cr_0212 | Carpathians | *L. l. carpathicus* | Poland | 22.43 | 49.71 | m | 19.88 | no |
| c_ll_mo_0184 | Mongolia | *L. l. wrangeli* | Mongolia | 108.64 | 48.38 | m | 7.3 | yes |
| c_ll_mo_0186 | Mongolia | *L. l. wrangeli* | Mongolia | 108.07 | 48.41 | f | 7.01 | yes |
| c_ll_mo_0188 | Mongolia | *L. l. wrangeli* | Mongolia | 110.49 | 48.62 | m | 6.31 | yes |
| c_ll_mo_0189 | Mongolia | *L. l. wrangeli* | Mongolia | 110.50 | 49.62 | m | 6.26 | yes |
| c_ll_mo_0181 | Mongolia | *L. l. wrangeli* | Mongolia | 101.05 | 43.23 | m | 7.08 | yes |
| c_ll_mo_0187 | Mongolia | *L. l. wrangeli* | Mongolia | 103.78 | 48.64 | f | 5.85 | yes |
| c_ll_mo_0190 | Mongolia | *L. l. wrangeli* | Mongolia | 108.27 | 47.40 | f | 7.58 | yes |
| c_ll_mo_0191 | Mongolia | *L. l. wrangeli* | Mongolia | 108.68 | 47.63 | m | 8.05 | yes |
| c_ll_ki_0090 | Kirov | *L. l. lynx* | Russia | 50.40 | 59.81 | f | 21.68 | yes |
| c_ll_ki_0091 | Kirov | *L. l. lynx* | Russia | 50.40 | 59.81 | m | 6.31 | yes |
| c_ll_ki_0092 | Kirov | *L. l. lynx* | Russia | 50.40 | 59.81 | f | 5.37 | yes |
| c_ll_ki_0093 | Kirov | *L. l. lynx* | Russia | 50.40 | 59.81 | m | 5.46 | yes |
| c_ll_ki_0094 | Kirov | *L. l. lynx* | Russia | 50.40 | 59.81 | m | 6.06 | yes |
| c_ll_ki_0095 | Kirov | *L. l. lynx* | Russia | 48.42 | 60.81 | m | 5.52 | yes |
| c_ll_ki_0096 | Kirov | *L. l. lynx* | Russia | 48.42 | 60.81 | m | 6.37 | yes |
| c_ll_ki_0097 | Kirov | *L. l. lynx* | Russia | 48.42 | 60.81 | f | 6.12 | yes |
| c_ll_ki_0098 | Kirov | *L. l. lynx* | Russia | 48.42 | 60.81 | f | 5.88 | yes |
| c_ll_ki_0099 | Kirov | *L. l. lynx* | Russia | 48.42 | 60.81 | f | 6.1 | yes |
| c_ll_ki_0100 | Kirov | *L. l. lynx* | Russia | 46.73 | 61.19 | m | 6.42 | yes |
| c_ll_ki_0101 | Kirov | *L. l. lynx* | Russia | 46.73 | 61.19 | f | 6.38 | yes |
| c_ll_ki_0102 | Kirov | *L. l. lynx* | Russia | 46.73 | 61.19 | m | 6.34 | yes |
| c_ll_la_0044 | Latvia | *L. l. lynx* | Latvia | 22.53 | 56.64 | m | 7.76 | yes |
| c_ll_la_0045 | Latvia | *L. l. lynx* | Latvia | 27.58 | 56.31 | f | 23.97 | yes |
| c_ll_la_0047 | Latvia | *L. l. lynx* | Latvia | 28.15 | 56.38 | f | 7.02 | yes |
| c_ll_la_0048 | Latvia | *L. l. lynx* | Latvia | 22.82 | 57.36 | f | 5.2 | yes |
| c_ll_la_0052 | Latvia | *L. l. lynx* | Latvia | 22.60 | 57.35 | m | 6.74 | yes |
| c_ll_la_0053 | Latvia | *L. l. lynx* | Latvia | 22.82 | 57.05 | f | 7.67 | yes |
| c_ll_la_0054 | Latvia | *L. l. lynx* | Latvia | 25.57 | 57.67 | m | 8.47 | yes |
| c_ll_no_0065 | Norway | *L. l. lynx* | Norway | 21.34 | 69.51 | f | 22.98 | no |
| c_ll_no_0075 | Norway | *L. l. lynx* | Norway | 12.36 | 64.51 | m | 5.59 | no |
| c_ll_no_0076 | Norway | *L. l. lynx* | Norway | 10.68 | 63.82 | m | 6 | no |
| c_ll_no_0077 | Norway | *L. l. lynx* | Norway | 10.95 | 63.57 | m | 5.1 | no |
| c_ll_no_0078 | Norway | *L. l. lynx* | Norway | 12.15 | 61.38 | m | 5.39 | no |
| c_ll_no_0079 | Norway | *L. l. lynx* | Norway | 8.41 | 59.40 | m | 5.11 | no |
| c_ll_no_0080 | Norway | *L. l. lynx* | Norway | 8.73 | 60.66 | f | 5.3 | no |
| c_ll_no_0081 | Norway | *L. l. lynx* | Norway | 8.97 | 60.27 | f | 5.59 | no |
| c_ll_no_0082 | Norway | *L. l. lynx* | Norway | 9.03 | 60.14 | m | 5.51 | no |
| c_ll_po_0001 | North-Eastern Poland | *L. l. lynx* | Poland | 23.60 | 52.95 | m | 6.73 | no |
| c_ll_po_0002 | North-Eastern Poland | *L. l. lynx* | Poland | 23.84 | 52.82 | f | 6.67 | no |
| c_ll_po_0003 | North-Eastern Poland | *L. l. lynx* | Poland | 23.72 | 52.80 | f | 6.49 | no |
| c_ll_po_0011 | North-Eastern Poland | *L. l. lynx* | Poland | 23.51 | 52.89 | m | 6.61 | no |
| c_ll_po_0014 | North-Eastern Poland | *L. l. lynx* | Poland | 23.95 | 52.77 | m | 6.61 | no |
| c_ll_po_0019 | North-Eastern Poland | *L. l. lynx* | Poland | 23.74 | 52.68 | m | 6.59 | no |
| c_ll_po_0105 | North-Eastern Poland | *L. l. lynx* | Poland | 23.47 | 53.09 | m | 5.44 | no |
| c_ll_po_0106 | North-Eastern Poland | *L. l. lynx* | Poland | 23.66 | 53.10 | f | 5.76 | no |
| c_ll_po_0150 | North-Eastern Poland | *L. l. lynx* | Poland | 23.60 | 52.76 | m | 23.99 | no |
| c_ll_tu_0153 | Tuva | *L. l. wrangeli* | Russia | 96.53 | 51.48 | f | 8.25 | yes |
| c_ll_tu_0154 | Tuva | *L. l. wrangeli* | Russia | 96.53 | 51.48 | f | 22.02 | yes |
| c_ll_tu_0157 | Tuva | *L. l. wrangeli* | Russia | 96.53 | 51.48 | m | 8.16 | yes |
| c_ll_tu_0158 | Tuva | *L. l. wrangeli* | Russia | 96.53 | 51.48 | f | 8.15 | yes |
| c_ll_tu_0159 | Tuva | *L. l. wrangeli* | Russia | 96.53 | 51.48 | f | 7.91 | yes |
| c_ll_tu_0165 | Tuva | *L. l. wrangeli* | Russia | 96.53 | 51.48 | f | 7.88 | yes |
| c_ll_tu_0166 | Tuva | *L. l. wrangeli* | Russia | 96.53 | 51.48 | m | 8.38 | yes |
| c_ll_ur_0194 | Urals | *L. l. lynx* | Russia | 59.64 | 56.05 | f | 11.29 | yes |
| c_ll_ur_0195 | Urals | *L. l. lynx* | Russia | 60.13 | 55.15 | m | 12.27 | yes |
| c_ll_ur_0196 | Urals | *L. l. lynx* | Russia | 60.13 | 55.15 | m | 12.91 | yes |
| c_ll_ur_0199 | Urals | *L. l. lynx* | Russia | 59.79 | 55.18 | m | 13.83 | yes |
| c_ll_ur_0200 | Urals | *L. l. lynx* | Russia | 59.00 | 55.00 | m | 13.33 | yes |
| c_ll_ur_0202 | Urals | *L. l. lynx* | Russia | 60.89 | 54.38 | f | 23.47 | yes |
| c_ll_ur_0203 | Urals | *L. l. lynx* | Russia | 57.36 | 55.00 | f | 13.79 | yes |
| c_ll_vl_0107 | Primosky Krai | *L. l. lynx* | Russia | 136.47 | 44.94 | f | 6.54 | yes |
| c_ll_vl_0108 | Primosky Krai | *L. l. lynx* | Russia | 135.58 | 45.29 | f | 11.58 | yes |
| c_ll_vl_0109 | Primosky Krai | *L. l. wrangeli* | Russia | 136.73 | 45.67 | m | 5.94 | yes |
| c_ll_vl_0110 | Primosky Krai | *L. l. wrangeli* | Russia | 132.91 | 49.01 | m | 7.08 | yes |
| c_ll_vl_0112 | Primosky Krai | *L. l. wrangeli* | Russia | 137.01 | 45.86 | f | 29.33 | yes |
| c_ll_vl_0113 | Primosky Krai | *L. l. wrangeli* | Russia | 137.01 | 45.86 | m | 8.75 | yes |
| c_ll_vl_0114 | Primosky Krai | *L. l. wrangeli* | Russia | 137.61 | 47.29 | f | 23.4 | yes |
| c_ll_vl_0128 | Primosky Krai | *L. l. wrangeli* | Russia | 137.43 | 45.91 | m | 8.29 | yes |
| c_ll_vl_0132 | Primosky Krai | *L. l. wrangeli* | Russia | 137.43 | 45.91 | m | 8.66 | yes |
| c_ll_vl_0137 | Primosky Krai | *L. l. wrangeli* | Russia | 133.46 | 51.39 | f | 25.47 | yes |
| c_ll_ya_0138 | Yakutia | *L. l. wrangeli* | Russia | 132.07 | 59.90 | m | 8.21 | yes |
| c_ll_ya_0139 | Yakutia | *L. l. wrangeli* | Russia | 129.20 | 61.19 | m | 8.28 | yes |
| c_ll_ya_0140 | Yakutia | *L. l. wrangeli* | Russia | 129.16 | 61.16 | m | 8.48 | yes |
| c_ll_ya_0141 | Yakutia | *L. l. wrangeli* | Russia | 136.18 | 66.89 | f | 23.6 | yes |
| c_ll_ya_0142 | Yakutia | *L. l. wrangeli* | Russia | 136.18 | 66.89 | m | 8.63 | yes |
| c_ll_ya_0143 | Yakutia | *L. l. wrangeli* | Russia | 136.18 | 66.89 | m | 8.14 | yes |
| c_ll_ya_0145 | Yakutia | *L. l. wrangeli* | Russia | 129.45 | 61.89 | m | 8.59 | yes |
| c_ll_ya_0146 | Yakutia | *L. l. wrangeli* | Russia | 130.68 | 60.78 | m | 23.58 | yes |
| c_ll_ya_0147 | Yakutia | *L. l. wrangeli* | Russia | 127.30 | 60.75 | f | 8.26 | yes |

**Table S2.** List of WorldClim bioclimatic variables used in this study, including the description provided by the WorldClim website and the abbreviation adopted in this study.

| **WorldClim variable** | **Meaning** | **Abbreviation** |
| --- | --- | --- |
| BIO1 | Annual Mean Temperature | T_mean_year |
| BIO2 | Mean Diurnal Range (Mean of monthly (max temp – min temp)) | T_range_day |
| BIO3 | Isothermality (BIO2/BIO7) (×100) | Iso_T |
| BIO4 | Temperature Seasonality (standard deviation ×100) | T_seasonality |
| BIO5 | Max Temperature of Warmest Month | T_max_warm |
| BIO6 | Min Temperature of Coldest Month | T_min_cold |
| BIO7 | Temperature Annual Range (BIO5-BIO6) | T_range_year |
| BIO8 | Mean Temperature of Wettest Quarter | T_wet_quart |
| BIO9 | Mean Temperature of Driest Quarter | T_dry_quart |
| BIO10 | Mean Temperature of Warmest Quarter | T_warm_quart |
| BIO11 | Mean Temperature of Coldest Quarter | T_cold_quart |
| BIO12 | Annual Precipitation | P_annual |
| BIO13 | Precipitation of Wettest Month | P_wet_month |
| BIO14 | Precipitation of Driest Month | P_dry_month |
| BIO15 | Precipitation Seasonality (Coefficient of Variation) | P_seasonality |
| BIO16 | Precipitation of Wettest Quarter | P_wet_quart |
| BIO17 | Precipitation of Driest Quarter | P_dry_quart |
| BIO18 | Precipitation of Warmest Quarter | P_warm_quart |
| BIO19 | Precipitation of Coldest Quarter | P_cold_quart |

**Table S3.** Summary of the forward model building, reporting variables, their adjusted r^2^, degrees of freedom (Df), AIC value, F value and p-value, in the order the were added to the null model.

| **Variable** | **Adjusted r2** | **Df** | **AIC** | **F** | **p-value** |
| --- | --- | --- | --- | --- | --- |
| **+ T_dry_quart** | 0.027322 | 1 | 950.94 | 2.9101 | 0.002 |
| **+ T_range_day** | 0.059092 | 1 | 949.62 | 3.2622 | 0.002 |
| **+ P_seasonality** | 0.088930 | 1 | 948.34 | 3.1616 | 0.002 |
| **+ P_wet_quart** | 0.098198 | 1 | 948.56 | 1.6680 | 0.004 |
| **+ P_wet_month** | 0.108735 | 1 | 948.67 | 1.7566 | 0.002 |
| **All variables** | 0.154015 |  |  |  |  |

**Table S4.** Summary of the analysis of variance partitioning. Inertia, r^2^, p-value and proportion of both explainable and total variance are reported for the full model, and the three distinct partial models. Confounded variance is the amount of the full model inertia that is not explainable by partial models.

| **Partial RDA models** | **Inertia** | ***r-squared*** | ***p-value*** | **Proportion of explainable Variance** | **Proportion of total Variance** |
| --- | --- | --- | --- | --- | --- |
| Full model: *F* ~ *clim*. + *geog*. + *struct*. | 252685 | 0.2615709 | 0.001*** | 1 | 0.26 |
| Pure climate: *F* ~ *clim*. \| (*geog*. + *struct*.) | 83295 | 0.086 | 0.001*** | 0.33 | 0.09 |
| Pure structure: *F* ~ *struct*. \| (*clim*. + *geog*.) | 55197 | 0.057 | 0.001*** | 0.22 | 0.06 |
| Pure geography: *F* ~ *geog*. \| (*clim*. + *struct*.) | 42160 | 0.044 | 0.001*** | 0.17 | 0.04 |
| Confounded climate/structure/geography | 72033 |  |  | 0.29 | 0.07 |
| Total unexplained | 713344 |  |  |  | 0.74 |
| Total inertia | 966029 |  |  |  | 1.00 |

**Table S5.** Summary of GenWin analysis for window boundary definition based on Bayes Factor values calculated by BayPass for each predictor environmental variable, and their overlap with candidate SNPs from RDA.

| **Variable** | **Number of Windows** | **Mean Length (bp)** | **Minimum Length (bp)** | **Maximum Length (bp)** | **SD Length (bp)** | **Windows with RDA SNPs** | **Max RDA SNPs in Window** |
| --- | --- | --- | --- | --- | --- | --- | --- |
| **T_dry_quart** | 1335 | 20951 | 10000 | 70000 | 10789 | 96 | 7 |
| **T_range_day** | 1342 | 20447 | 10000 | 70000 | 10295 | 108 | 8 |
| **P_seasonality** | 1329 | 21128 | 10000 | 70000 | 10567 | 54 | 5 |
| **P_wet_quart** | 1338 | 20440 | 10000 | 80000 | 10458 | 68 | 4 |
| **Mean_snow_days** | 1325 | 20943 | 10000 | 90000 | 10712 | 279 | 9 |

**Table S6.** List of candidate windows from univariate analysis overlapping with at least one candidate SNP from the multivariate analysis. Genomic position in terms of scaffold name, window start and end coordinates are reported, together with the variable from the univariate analysis from which the window was found, its support (W-stat), the number of candidate RDA SNPs found within the window and the genes present in the window.

| **Chromosome** | **Window Start** | **Window Stop** | **Variable** | **W-stat** | **RDA SNPs** | **Genes** |
| --- | --- | --- | --- | --- | --- | --- |
| scaffold_11_arrow_ctg1 | 1215000 | 1225000 | bio15 | 8.57 | 1 | DPYSL4\|JAKMIP3\|STK32C |
| scaffold_11_arrow_ctg1 | 22485000 | 22545000 | bio15 | 7.30 | 1 | SORCS1 |
| scaffold_11_arrow_ctg1 | 23425000 | 23455000 | bio15 | 15.78 | 1 | NA |
| scaffold_11_arrow_ctg1 | 23455000 | 23495000 | bio15 | 11.08 | 1 | NA |
| scaffold_11_arrow_ctg1 | 86545000 | 86565000 | bio15 | 7.90 | 1 | NA |
| Super_Scaffold_7 | 39695000 | 39725000 | bio15 | 11.37 | 1 | CPQ |
| Super_Scaffold_7 | 43625000 | 43665000 | bio15 | 8.91 | 2 | NA |
| Super_Scaffold_7 | 64185000 | 64225000 | bio15 | 8.24 | 1 | NA |
| Super_Scaffold_7 | 65305000 | 65325000 | bio15 | 9.59 | 1 | CPA6 |
| Super_Scaffold_7 | 74385000 | 74415000 | bio15 | 7.49 | 1 | NA |
| Super_Scaffold_8 | 11605000 | 11645000 | bio15 | 7.29 | 2 | FXN |
| Super_Scaffold_8 | 76275000 | 76295000 | bio15 | 7.56 | 1 | ASTN2 |
| scaffold_17_arrow_ctg1 | 3855000 | 3875000 | bio15 | 10.64 | 1 | SMYD3 |
| scaffold_17_arrow_ctg1 | 42315000 | 42335000 | bio15 | 8.10 | 1 | PLEKHA6 |
| scaffold_17_arrow_ctg1 | 46405000 | 46415000 | bio15 | 7.52 | 1 | NA |
| scaffold_17_arrow_ctg1 | 46615000 | 46635000 | bio15 | 11.63 | 1 | NA |
| Super_Scaffold_4 | 1195000 | 1215000 | bio15 | 10.38 | 1 | PEX10\|PLCH2\|RER1 |
| Super_Scaffold_4 | 73535000 | 73555000 | bio15 | 8.12 | 3 | NA |
| Super_Scaffold_4 | 73565000 | 73575000 | bio15 | 15.66 | 1 | NA |
| Super_Scaffold_4 | 73575000 | 73595000 | bio15 | 9.01 | 1 | NA |
| Super_Scaffold_4 | 122855000 | 122875000 | bio15 | 7.42 | 1 | NA |
| Super_Scaffold_4 | 131155000 | 131195000 | bio15 | 8.65 | 1 | LRP1B |
| Super_Scaffold_4 | 174855000 | 174895000 | bio15 | 8.10 | 2 | NA |
| Super_Scaffold_5 | 29485000 | 29505000 | bio15 | 7.30 | 1 | PTPRG |
| scaffold_2_arrow_ctg1 | 47545000 | 47555000 | bio15 | 9.47 | 2 | NA |
| scaffold_2_arrow_ctg1 | 58355000 | 58405000 | bio15 | 7.62 | 2 | NA |
| scaffold_2_arrow_ctg1 | 66245000 | 66275000 | bio15 | 7.81 | 1 | FSTL5 |
| scaffold_2_arrow_ctg1 | 72745000 | 72765000 | bio15 | 8.29 | 2 | NA |
| scaffold_2_arrow_ctg1 | 72765000 | 72785000 | bio15 | 8.28 | 4 | NA |
| scaffold_2_arrow_ctg1 | 136405000 | 136445000 | bio15 | 8.41 | 5 | WDFY3 |
| scaffold_2_arrow_ctg1 | 167465000 | 167485000 | bio15 | 8.48 | 1 | GABRG1 |
| scaffold_2_arrow_ctg1 | 170185000 | 170195000 | bio15 | 7.29 | 1 | GRXCR1 |
| scaffold_2_arrow_ctg1 | 171115000 | 171135000 | bio15 | 7.49 | 1 | PHOX2B |
| Super_Scaffold_1 | 217255000 | 217295000 | bio15 | 9.41 | 1 | NA |
| Super_Scaffold_3 | 45885000 | 45935000 | bio15 | 10.86 | 1 | PTCHD4 |
| Super_Scaffold_3 | 84185000 | 84205000 | bio15 | 11.40 | 1 | NA |
| Super_Scaffold_3 | 86565000 | 86585000 | bio15 | 8.46 | 1 | NA |
| Super_Scaffold_3 | 86585000 | 86615000 | bio15 | 13.95 | 1 | NA |
| Super_Scaffold_2 | 195000 | 215000 | bio15 | 7.46 | 1 | HYKK\|IREB2 |
| Super_Scaffold_2 | 4485000 | 4555000 | bio15 | 8.23 | 1 | ALPK3\|SEC11A\|ZNF592 |
| Super_Scaffold_2 | 13365000 | 13375000 | bio15 | 7.26 | 1 | NA |
| Super_Scaffold_2 | 34165000 | 34205000 | bio15 | 12.98 | 1 | THSD4 |
| Super_Scaffold_2 | 34205000 | 34225000 | bio15 | 7.90 | 1 | THSD4 |
| Super_Scaffold_2 | 69855000 | 69875000 | bio15 | 7.98 | 2 | LOC115516166\|LOC115517158\|LOC115517160 |
| Super_Scaffold_2 | 104285000 | 104295000 | bio15 | 9.01 | 1 | AP5M1\|EXOC5 |
| Super_Scaffold_13 | 8225000 | 8245000 | bio15 | 8.75 | 2 | NA |
| Super_Scaffold_13 | 80215000 | 80275000 | bio15 | 10.83 | 1 | GTSF1\|NCKAP1L\|PDE1B\|PPP1R1A |
| Super_Scaffold_12 | 13335000 | 13365000 | bio15 | 9.69 | 2 | RBMS3 |
| Super_Scaffold_12 | 13425000 | 13445000 | bio15 | 9.08 | 1 | RBMS3 |
| Super_Scaffold_12 | 18975000 | 19015000 | bio15 | 9.95 | 3 | LOC115523127 |
| Super_Scaffold_12 | 21145000 | 21175000 | bio15 | 8.31 | 1 | NA |
| Super_Scaffold_12 | 35855000 | 35885000 | bio15 | 9.02 | 2 | CLSTN2 |
| Super_Scaffold_12 | 35885000 | 35915000 | bio15 | 14.50 | 1 | CLSTN2 |
| Super_Scaffold_14 | 61235000 | 61245000 | bio15 | 7.53 | 1 | LOC115525358\|LOC115525566\|LOC115526263\|LOC115526264 |
| scaffold_11_arrow_ctg1 | 1495000 | 1515000 | bio16 | 8.59 | 1 | NA |
| scaffold_11_arrow_ctg1 | 39035000 | 39045000 | bio16 | 8.25 | 1 | NA |
| scaffold_11_arrow_ctg1 | 39045000 | 39065000 | bio16 | 8.14 | 4 | NA |
| scaffold_11_arrow_ctg1 | 42435000 | 42465000 | bio16 | 9.17 | 1 | BMPR1A\|MMRN2\|SNCG |
| scaffold_11_arrow_ctg1 | 78765000 | 78785000 | bio16 | 9.18 | 1 | ACTA2\|STAMBPL1 |
| scaffold_11_arrow_ctg1 | 82085000 | 82105000 | bio16 | 12.64 | 1 | 1.00 DKK |
| scaffold_11_arrow_ctg1 | 83445000 | 83455000 | bio16 | 10.76 | 1 | PCDH15 |
| scaffold_11_arrow_ctg1 | 84575000 | 84595000 | bio16 | 8.22 | 1 | NA |
| Super_Scaffold_7 | 16065000 | 16075000 | bio16 | 8.65 | 1 | FER1L6 |
| Super_Scaffold_8 | 3175000 | 3195000 | bio16 | 7.55 | 1 | LOC115499494 |
| Super_Scaffold_8 | 27625000 | 27665000 | bio16 | 7.60 | 1 | NA |
| Super_Scaffold_8 | 35595000 | 35625000 | bio16 | 22.92 | 1 | PTPRD |
| scaffold_17_arrow_ctg1 | 17635000 | 17655000 | bio16 | 13.78 | 1 | 1 COP |
| scaffold_17_arrow_ctg1 | 17875000 | 17885000 | bio16 | 19.25 | 1 | 1 COP |
| scaffold_17_arrow_ctg1 | 17905000 | 17915000 | bio16 | 17.74 | 1 | 1 COP |
| scaffold_17_arrow_ctg1 | 18005000 | 18015000 | bio16 | 28.93 | 1 | NA |
| scaffold_17_arrow_ctg1 | 18035000 | 18045000 | bio16 | 30.76 | 1 | NA |
| scaffold_17_arrow_ctg1 | 18115000 | 18135000 | bio16 | 27.35 | 1 | NA |
| scaffold_17_arrow_ctg1 | 29045000 | 29065000 | bio16 | 9.98 | 1 | PTPN14\|SMYD2 |
| scaffold_17_arrow_ctg1 | 46615000 | 46635000 | bio16 | 21.40 | 1 | NA |
| scaffold_17_arrow_ctg1 | 46695000 | 46715000 | bio16 | 11.59 | 1 | NA |
| scaffold_17_arrow_ctg1 | 59345000 | 59355000 | bio16 | 8.85 | 1 | NA |
| scaffold_18_arrow_ctg1 | 8145000 | 8165000 | bio16 | 8.73 | 1 | NA |
| Super_Scaffold_4 | 22245000 | 22275000 | bio16 | 9.85 | 1 | NA |
| Super_Scaffold_4 | 45305000 | 45335000 | bio16 | 7.60 | 1 | NA |
| Super_Scaffold_4 | 45695000 | 45725000 | bio16 | 7.96 | 2 | FYB2 |
| Super_Scaffold_4 | 131165000 | 131195000 | bio16 | 7.77 | 1 | LRP1B |
| Super_Scaffold_4 | 131715000 | 131745000 | bio16 | 11.89 | 1 | LRP1B |
| Super_Scaffold_4 | 211675000 | 211685000 | bio16 | 15.85 | 1 | DNER |
| Super_Scaffold_4 | 215095000 | 215125000 | bio16 | 8.03 | 2 | NA |
| Super_Scaffold_5 | 49185000 | 49255000 | bio16 | 9.57 | 1 | GRM7 |
| Super_Scaffold_5 | 133515000 | 133555000 | bio16 | 8.43 | 2 | NA |
| scaffold_2_arrow_ctg1 | 11885000 | 11915000 | bio16 | 12.75 | 3 | NA |
| scaffold_2_arrow_ctg1 | 13935000 | 13965000 | bio16 | 9.73 | 1 | WWC2 |
| scaffold_2_arrow_ctg1 | 42685000 | 42705000 | bio16 | 15.27 | 1 | IDO1\|IDO2 |
| scaffold_2_arrow_ctg1 | 42755000 | 42775000 | bio16 | 11.59 | 1 | LOC115511986 |
| scaffold_2_arrow_ctg1 | 42775000 | 42785000 | bio16 | 9.21 | 1 | LOC115511986 |
| scaffold_2_arrow_ctg1 | 42795000 | 42835000 | bio16 | 13.27 | 3 | LOC115511986 |
| scaffold_2_arrow_ctg1 | 42835000 | 42865000 | bio16 | 8.51 | 4 | LOC115511986 |
| scaffold_2_arrow_ctg1 | 42885000 | 42905000 | bio16 | 10.73 | 2 | NA |
| scaffold_2_arrow_ctg1 | 42915000 | 42925000 | bio16 | 11.87 | 2 | NA |
| scaffold_2_arrow_ctg1 | 42995000 | 43015000 | bio16 | 14.30 | 1 | ADAM2 |
| scaffold_2_arrow_ctg1 | 167165000 | 167195000 | bio16 | 18.69 | 1 | GABRA2 |
| scaffold_2_arrow_ctg1 | 170145000 | 170165000 | bio16 | 9.35 | 1 | GRXCR1 |
| Super_Scaffold_1 | 30905000 | 30935000 | bio16 | 8.78 | 2 | NA |
| Super_Scaffold_1 | 33145000 | 33165000 | bio16 | 12.68 | 1 | NA |
| Super_Scaffold_1 | 65615000 | 65635000 | bio16 | 9.94 | 1 | GPC6 |
| Super_Scaffold_1 | 137505000 | 137535000 | bio16 | 9.01 | 1 | NA |
| Super_Scaffold_1 | 229145000 | 229155000 | bio16 | 8.12 | 1 | DNAH5 |
| Super_Scaffold_3 | 38965000 | 38995000 | bio16 | 9.08 | 1 | NA |
| Super_Scaffold_2 | 97285000 | 97305000 | bio16 | 20.71 | 1 | NA |
| Super_Scaffold_2 | 97355000 | 97385000 | bio16 | 11.75 | 1 | DNAAF2\|LRR1\|MGAT2\|POLE2\|RPL36AL\|RPS29 |
| Super_Scaffold_13 | 10475000 | 10495000 | bio16 | 8.32 | 1 | CELF2 |
| Super_Scaffold_13 | 86105000 | 86115000 | bio16 | 10.58 | 1 | NA |
| Super_Scaffold_13 | 135535000 | 135565000 | bio16 | 9.63 | 2 | SCUBE1 |
| Super_Scaffold_12 | 3565000 | 3575000 | bio16 | 14.10 | 2 | NA |
| Super_Scaffold_12 | 6355000 | 6365000 | bio16 | 13.54 | 1 | XYLB |
| Super_Scaffold_12 | 13415000 | 13465000 | bio16 | 13.39 | 1 | RBMS3 |
| Super_Scaffold_12 | 46565000 | 46595000 | bio16 | 15.30 | 1 | NA |
| Super_Scaffold_12 | 139155000 | 139175000 | bio16 | 18.21 | 1 | NA |
| Super_Scaffold_12 | 139225000 | 139255000 | bio16 | 10.24 | 1 | NA |
| Super_Scaffold_14 | 38575000 | 38625000 | bio16 | 7.60 | 1 | NA |
| Super_Scaffold_14 | 38655000 | 38675000 | bio16 | 9.63 | 2 | SESN3 |
| Super_Scaffold_14 | 56665000 | 56705000 | bio16 | 10.42 | 1 | MAP6 |
| Super_Scaffold_14 | 61215000 | 61245000 | bio16 | 13.96 | 1 | LOC115525358\|LOC115525539\|LOC115525566\|LOC115526263\|LOC115526264 |
| Super_Scaffold_14 | 67955000 | 67965000 | bio16 | 7.87 | 1 | GALNT18 |
| Super_Scaffold_14 | 100075000 | 100115000 | bio16 | 9.62 | 2 | NUP160 |
| Super_Scaffold_14 | 104715000 | 104735000 | bio16 | 20.63 | 1 | LOC115524657\|LOC115524658\|LOC115524659\|LOC115524660\|LOC115526441 |
| scaffold_11_arrow_ctg1 | 3205000 | 3225000 | bio2 | 8.22 | 1 | MGMT |
| scaffold_11_arrow_ctg1 | 10785000 | 10805000 | bio2 | 14.54 | 1 | NA |
| scaffold_11_arrow_ctg1 | 19195000 | 19215000 | bio2 | 8.64 | 2 | NA |
| scaffold_11_arrow_ctg1 | 23445000 | 23465000 | bio2 | 19.64 | 1 | NA |
| scaffold_11_arrow_ctg1 | 23485000 | 23505000 | bio2 | 8.41 | 2 | NA |
| scaffold_11_arrow_ctg1 | 37845000 | 37865000 | bio2 | 7.48 | 1 | RASGEF1A |
| scaffold_11_arrow_ctg1 | 60925000 | 60945000 | bio2 | 9.40 | 2 | LOC115527552 |
| scaffold_11_arrow_ctg1 | 86545000 | 86565000 | bio2 | 8.52 | 1 | NA |
| Super_Scaffold_6 | 39225000 | 39245000 | bio2 | 9.75 | 1 | NA |
| Super_Scaffold_6 | 58545000 | 58585000 | bio2 | 12.83 | 1 | PTPRM |
| Super_Scaffold_6 | 90615000 | 90655000 | bio2 | 7.70 | 1 | GLT1D1\|SLC15A4 |
| Super_Scaffold_6 | 90865000 | 90895000 | bio2 | 8.52 | 2 | TMEM132D |
| Super_Scaffold_7 | 39705000 | 39715000 | bio2 | 9.26 | 1 | CPQ |
| Super_Scaffold_7 | 43655000 | 43675000 | bio2 | 8.33 | 2 | NA |
| Super_Scaffold_7 | 65305000 | 65325000 | bio2 | 7.49 | 1 | CPA6 |
| Super_Scaffold_7 | 74385000 | 74415000 | bio2 | 13.74 | 1 | NA |
| Super_Scaffold_8 | 11605000 | 11645000 | bio2 | 9.73 | 2 | FXN |
| scaffold_17_arrow_ctg1 | 17465000 | 17495000 | bio2 | 10.32 | 1 | TNR |
| scaffold_17_arrow_ctg1 | 42195000 | 42205000 | bio2 | 8.23 | 1 | ETNK2\|SOX13 |
| scaffold_17_arrow_ctg1 | 42285000 | 42325000 | bio2 | 8.89 | 1 | GOLT1A\|KISS1\|PLEKHA6 |
| scaffold_17_arrow_ctg1 | 46615000 | 46635000 | bio2 | 25.24 | 1 | NA |
| scaffold_17_arrow_ctg1 | 46705000 | 46715000 | bio2 | 9.15 | 1 | NA |
| scaffold_18_arrow_ctg1 | 8665000 | 8675000 | bio2 | 8.28 | 2 | NA |
| scaffold_18_arrow_ctg1 | 54875000 | 54885000 | bio2 | 8.50 | 2 | LLGL2\|LOC115501833\|LOC115501837 |
| scaffold_18_arrow_ctg1 | 54885000 | 54905000 | bio2 | 9.06 | 1 | LLGL2\|LOC115501833\|LOC115501837\|RECQL5 |
| Super_Scaffold_4 | 3515000 | 3555000 | bio2 | 10.84 | 8 | NA |
| Super_Scaffold_4 | 12705000 | 12745000 | bio2 | 8.77 | 4 | IGSF21 |
| Super_Scaffold_4 | 73535000 | 73555000 | bio2 | 13.90 | 3 | NA |
| Super_Scaffold_4 | 73565000 | 73575000 | bio2 | 17.91 | 1 | NA |
| Super_Scaffold_4 | 73575000 | 73595000 | bio2 | 8.71 | 1 | NA |
| Super_Scaffold_4 | 131165000 | 131185000 | bio2 | 7.95 | 1 | LRP1B |
| Super_Scaffold_4 | 182935000 | 182975000 | bio2 | 7.89 | 1 | ANKRD44 |
| Super_Scaffold_4 | 212055000 | 212075000 | bio2 | 7.47 | 1 | LOC115521092\|LOC115521897 |
| Super_Scaffold_5 | 15485000 | 15495000 | bio2 | 7.43 | 1 | LZTFL1\|SLC6A20 |
| Super_Scaffold_5 | 29175000 | 29205000 | bio2 | 8.16 | 2 | PTPRG |
| Super_Scaffold_5 | 141415000 | 141445000 | bio2 | 8.92 | 2 | GPR37 |
| Super_Scaffold_5 | 168185000 | 168225000 | bio2 | 8.50 | 1 | PTPRN2 |
| scaffold_2_arrow_ctg1 | 47545000 | 47555000 | bio2 | 10.09 | 2 | NA |
| scaffold_2_arrow_ctg1 | 66245000 | 66275000 | bio2 | 10.90 | 1 | FSTL5 |
| scaffold_2_arrow_ctg1 | 72745000 | 72765000 | bio2 | 14.41 | 2 | NA |
| scaffold_2_arrow_ctg1 | 72765000 | 72785000 | bio2 | 16.77 | 4 | NA |
| scaffold_2_arrow_ctg1 | 72785000 | 72795000 | bio2 | 9.69 | 2 | NA |
| scaffold_2_arrow_ctg1 | 109275000 | 109295000 | bio2 | 7.92 | 2 | LOC115512302 |
| scaffold_2_arrow_ctg1 | 139485000 | 139515000 | bio2 | 16.84 | 1 | NA |
| scaffold_2_arrow_ctg1 | 139595000 | 139605000 | bio2 | 9.53 | 1 | PRKG2 |
| scaffold_2_arrow_ctg1 | 168485000 | 168515000 | bio2 | 8.04 | 3 | NA |
| scaffold_2_arrow_ctg1 | 168515000 | 168535000 | bio2 | 12.52 | 1 | NA |
| scaffold_2_arrow_ctg1 | 179855000 | 179915000 | bio2 | 9.53 | 2 | NA |
| scaffold_2_arrow_ctg1 | 194615000 | 194635000 | bio2 | 8.64 | 1 | CC2D2A\|FBXL5 |
| Super_Scaffold_1 | 6685000 | 6705000 | bio2 | 7.96 | 1 | USP12 |
| Super_Scaffold_1 | 35125000 | 35145000 | bio2 | 9.62 | 1 | NA |
| Super_Scaffold_1 | 101155000 | 101185000 | bio2 | 10.26 | 1 | PPIC\|SNX24 |
| Super_Scaffold_1 | 103005000 | 103045000 | bio2 | 7.62 | 1 | NA |
| Super_Scaffold_1 | 103445000 | 103465000 | bio2 | 9.81 | 1 | NA |
| Super_Scaffold_1 | 178885000 | 178905000 | bio2 | 7.97 | 1 | KCNIP1 |
| Super_Scaffold_1 | 232805000 | 232825000 | bio2 | 7.99 | 2 | SEMA5A |
| Super_Scaffold_3 | 84165000 | 84185000 | bio2 | 9.09 | 1 | NA |
| Super_Scaffold_3 | 84185000 | 84205000 | bio2 | 9.31 | 1 | NA |
| Super_Scaffold_3 | 101025000 | 101055000 | bio2 | 13.20 | 1 | REV3L |
| Super_Scaffold_3 | 123305000 | 123315000 | bio2 | 8.42 | 1 | PDE7B |
| Super_Scaffold_2 | 34195000 | 34205000 | bio2 | 8.79 | 1 | THSD4 |
| Super_Scaffold_2 | 60445000 | 60475000 | bio2 | 11.25 | 1 | STARD9 |
| Super_Scaffold_2 | 69855000 | 69875000 | bio2 | 12.41 | 2 | LOC115516166\|LOC115517158\|LOC115517160 |
| Super_Scaffold_2 | 97285000 | 97305000 | bio2 | 9.60 | 1 | NA |
| Super_Scaffold_2 | 132825000 | 132875000 | bio2 | 8.67 | 1 | KCNK13\|LOC115516100\|TDP1 |
| Super_Scaffold_2 | 134665000 | 134695000 | bio2 | 7.81 | 1 | NA |
| Super_Scaffold_13 | 4375000 | 4385000 | bio2 | 7.51 | 1 | NA |
| Super_Scaffold_13 | 4945000 | 4995000 | bio2 | 9.87 | 2 | GDI2\|TASOR2 |
| Super_Scaffold_13 | 8225000 | 8245000 | bio2 | 9.58 | 2 | NA |
| Super_Scaffold_13 | 27695000 | 27715000 | bio2 | 10.41 | 2 | SVIL |
| Super_Scaffold_13 | 38705000 | 38715000 | bio2 | 7.45 | 1 | PARP11 |
| Super_Scaffold_13 | 53815000 | 53845000 | bio2 | 11.39 | 1 | NA |
| Super_Scaffold_13 | 54125000 | 54135000 | bio2 | 10.18 | 1 | NA |
| Super_Scaffold_13 | 68235000 | 68265000 | bio2 | 8.76 | 3 | PDZRN4 |
| Super_Scaffold_13 | 68265000 | 68285000 | bio2 | 12.18 | 2 | PDZRN4 |
| Super_Scaffold_13 | 68285000 | 68295000 | bio2 | 13.24 | 1 | PDZRN4 |
| Super_Scaffold_13 | 68295000 | 68305000 | bio2 | 13.16 | 2 | PDZRN4 |
| Super_Scaffold_13 | 68305000 | 68335000 | bio2 | 16.31 | 4 | PDZRN4 |
| Super_Scaffold_13 | 68335000 | 68345000 | bio2 | 11.02 | 1 | PDZRN4 |
| Super_Scaffold_13 | 68375000 | 68385000 | bio2 | 8.94 | 2 | PDZRN4 |
| Super_Scaffold_13 | 68385000 | 68415000 | bio2 | 17.07 | 3 | PDZRN4 |
| Super_Scaffold_13 | 68415000 | 68425000 | bio2 | 13.90 | 2 | PDZRN4 |
| Super_Scaffold_13 | 68425000 | 68435000 | bio2 | 9.51 | 1 | PDZRN4 |
| Super_Scaffold_13 | 80245000 | 80275000 | bio2 | 7.81 | 1 | NCKAP1L\|PDE1B\|PPP1R1A |
| Super_Scaffold_9 | 2315000 | 2325000 | bio2 | 9.67 | 2 | CDH4 |
| Super_Scaffold_9 | 5965000 | 5995000 | bio2 | 10.75 | 3 | NA |
| Super_Scaffold_9 | 45165000 | 45185000 | bio2 | 7.74 | 1 | RALGAPA2 |
| Super_Scaffold_9 | 75685000 | 75705000 | bio2 | 8.04 | 1 | NA |
| Super_Scaffold_9 | 117855000 | 117865000 | bio2 | 8.30 | 1 | MRPL33\|RBKS |
| Super_Scaffold_12 | 4405000 | 4425000 | bio2 | 7.37 | 2 | NA |
| Super_Scaffold_12 | 6335000 | 6365000 | bio2 | 11.04 | 1 | XYLB |
| Super_Scaffold_12 | 18985000 | 19005000 | bio2 | 13.80 | 2 | LOC115523127 |
| Super_Scaffold_12 | 19015000 | 19025000 | bio2 | 7.59 | 1 | LOC115523127 |
| Super_Scaffold_12 | 19035000 | 19075000 | bio2 | 11.22 | 6 | LOC115523127 |
| Super_Scaffold_12 | 19085000 | 19105000 | bio2 | 8.41 | 3 | LOC115523127 |
| Super_Scaffold_12 | 19105000 | 19115000 | bio2 | 12.64 | 2 | NA |
| Super_Scaffold_12 | 35855000 | 35885000 | bio2 | 8.67 | 2 | CLSTN2 |
| Super_Scaffold_12 | 35885000 | 35915000 | bio2 | 11.20 | 1 | CLSTN2 |
| Super_Scaffold_12 | 84445000 | 84485000 | bio2 | 8.25 | 5 | NA |
| Super_Scaffold_12 | 120625000 | 120655000 | bio2 | 13.96 | 2 | CADM2 |
| Super_Scaffold_14 | 24165000 | 24195000 | bio2 | 9.61 | 3 | NA |
| Super_Scaffold_14 | 38665000 | 38695000 | bio2 | 10.58 | 1 | SESN3 |
| Super_Scaffold_14 | 51625000 | 51665000 | bio2 | 11.54 | 1 | NA |
| Super_Scaffold_14 | 51665000 | 51685000 | bio2 | 9.03 | 1 | NA |
| Super_Scaffold_14 | 51685000 | 51705000 | bio2 | 9.63 | 2 | NA |
| Super_Scaffold_14 | 51755000 | 51785000 | bio2 | 8.75 | 2 | NA |
| Super_Scaffold_14 | 61235000 | 61245000 | bio2 | 9.81 | 1 | LOC115525358\|LOC115525566\|LOC115526263\|LOC115526264 |
| Super_Scaffold_14 | 104825000 | 104845000 | bio2 | 8.19 | 2 | LOC115525320\|LOC115526000\|LOC115526443 |
| scaffold_11_arrow_ctg1 | 3355000 | 3375000 | bio9 | 7.32 | 1 | NA |
| scaffold_11_arrow_ctg1 | 10735000 | 10805000 | bio9 | 10.65 | 2 | WDR11 |
| scaffold_11_arrow_ctg1 | 36125000 | 36145000 | bio9 | 12.59 | 1 | NA |
| scaffold_11_arrow_ctg1 | 60905000 | 60935000 | bio9 | 17.49 | 2 | LOC115527552 |
| scaffold_11_arrow_ctg1 | 60935000 | 60945000 | bio9 | 20.44 | 1 | NA |
| scaffold_11_arrow_ctg1 | 60945000 | 60955000 | bio9 | 16.76 | 1 | NA |
| scaffold_11_arrow_ctg1 | 63075000 | 63105000 | bio9 | 11.22 | 4 | NA |
| scaffold_11_arrow_ctg1 | 82065000 | 82095000 | bio9 | 7.92 | 1 | 1.00 DKK |
| scaffold_11_arrow_ctg1 | 82155000 | 82185000 | bio9 | 8.83 | 1 | NA |
| scaffold_11_arrow_ctg1 | 82185000 | 82195000 | bio9 | 7.76 | 1 | NA |
| Super_Scaffold_6 | 90615000 | 90645000 | bio9 | 7.85 | 1 | GLT1D1\|SLC15A4 |
| Super_Scaffold_7 | 45615000 | 45645000 | bio9 | 13.51 | 3 | NA |
| Super_Scaffold_7 | 47315000 | 47335000 | bio9 | 14.66 | 1 | NA |
| Super_Scaffold_8 | 8535000 | 8585000 | bio9 | 13.36 | 1 | KCNV2 |
| Super_Scaffold_8 | 8705000 | 8725000 | bio9 | 8.37 | 1 | NA |
| Super_Scaffold_8 | 35605000 | 35625000 | bio9 | 13.61 | 1 | PTPRD |
| scaffold_17_arrow_ctg1 | 5595000 | 5625000 | bio9 | 7.82 | 2 | NA |
| scaffold_17_arrow_ctg1 | 17645000 | 17655000 | bio9 | 10.00 | 1 | 1 COP |
| scaffold_17_arrow_ctg1 | 17875000 | 17885000 | bio9 | 17.04 | 1 | 1 COP |
| scaffold_17_arrow_ctg1 | 17905000 | 17925000 | bio9 | 19.54 | 1 | 1 COP |
| scaffold_17_arrow_ctg1 | 18005000 | 18015000 | bio9 | 25.39 | 1 | NA |
| scaffold_17_arrow_ctg1 | 18035000 | 18045000 | bio9 | 26.85 | 1 | NA |
| scaffold_17_arrow_ctg1 | 18115000 | 18125000 | bio9 | 10.53 | 1 | NA |
| scaffold_17_arrow_ctg1 | 41765000 | 41775000 | bio9 | 7.53 | 2 | NA |
| scaffold_17_arrow_ctg1 | 46615000 | 46635000 | bio9 | 16.46 | 1 | NA |
| scaffold_17_arrow_ctg1 | 55905000 | 55915000 | bio9 | 10.26 | 1 | NA |
| scaffold_17_arrow_ctg1 | 55915000 | 55955000 | bio9 | 9.25 | 2 | NA |
| scaffold_18_arrow_ctg1 | 54865000 | 54895000 | bio9 | 8.83 | 3 | LLGL2\|LOC115501833\|LOC115501837\|TSEN54 |
| Super_Scaffold_11 | 9945000 | 9965000 | bio9 | 8.97 | 3 | VAT1L |
| Super_Scaffold_11 | 32355000 | 32365000 | bio9 | 8.91 | 1 | NA |
| Super_Scaffold_4 | 22245000 | 22295000 | bio9 | 9.74 | 4 | NA |
| Super_Scaffold_4 | 22295000 | 22315000 | bio9 | 8.97 | 2 | NA |
| Super_Scaffold_4 | 73535000 | 73555000 | bio9 | 13.97 | 3 | NA |
| Super_Scaffold_4 | 73565000 | 73575000 | bio9 | 13.85 | 1 | NA |
| Super_Scaffold_4 | 73575000 | 73595000 | bio9 | 8.44 | 1 | NA |
| Super_Scaffold_4 | 73595000 | 73625000 | bio9 | 8.64 | 1 | NA |
| Super_Scaffold_4 | 93625000 | 93675000 | bio9 | 10.12 | 2 | KCNC4\|SLC6A17 |
| Super_Scaffold_4 | 93705000 | 93735000 | bio9 | 7.63 | 1 | NA |
| Super_Scaffold_4 | 131135000 | 131155000 | bio9 | 8.14 | 1 | LRP1B |
| Super_Scaffold_4 | 131165000 | 131175000 | bio9 | 12.28 | 1 | LRP1B |
| Super_Scaffold_4 | 131705000 | 131735000 | bio9 | 9.80 | 1 | LRP1B |
| Super_Scaffold_4 | 179895000 | 179905000 | bio9 | 8.22 | 1 | NA |
| Super_Scaffold_4 | 209995000 | 210005000 | bio9 | 7.67 | 1 | AGFG1 |
| Super_Scaffold_4 | 210005000 | 210035000 | bio9 | 7.45 | 4 | AGFG1 |
| Super_Scaffold_4 | 212055000 | 212075000 | bio9 | 8.14 | 1 | LOC115521092\|LOC115521897 |
| Super_Scaffold_4 | 215095000 | 215125000 | bio9 | 7.90 | 2 | NA |
| Super_Scaffold_5 | 29185000 | 29205000 | bio9 | 8.90 | 1 | PTPRG |
| Super_Scaffold_5 | 64345000 | 64355000 | bio9 | 11.64 | 1 | VWC2 |
| scaffold_2_arrow_ctg1 | 47535000 | 47555000 | bio9 | 10.15 | 2 | NA |
| scaffold_2_arrow_ctg1 | 164065000 | 164075000 | bio9 | 8.01 | 2 | USP46 |
| scaffold_2_arrow_ctg1 | 167165000 | 167195000 | bio9 | 8.82 | 1 | GABRA2 |
| scaffold_2_arrow_ctg1 | 174005000 | 174025000 | bio9 | 8.02 | 2 | NA |
| scaffold_2_arrow_ctg1 | 200035000 | 200085000 | bio9 | 9.03 | 1 | NA |
| Super_Scaffold_1 | 6465000 | 6475000 | bio9 | 8.35 | 2 | GPR12 |
| Super_Scaffold_1 | 30895000 | 30915000 | bio9 | 9.55 | 3 | NA |
| Super_Scaffold_1 | 33145000 | 33165000 | bio9 | 7.27 | 1 | NA |
| Super_Scaffold_1 | 49375000 | 49405000 | bio9 | 8.95 | 1 | NA |
| Super_Scaffold_1 | 190075000 | 190135000 | bio9 | 15.66 | 1 | NA |
| Super_Scaffold_1 | 198095000 | 198125000 | bio9 | 7.98 | 1 | SH3TC2 |
| Super_Scaffold_1 | 198125000 | 198145000 | bio9 | 9.88 | 4 | NA |
| Super_Scaffold_3 | 38995000 | 39025000 | bio9 | 9.55 | 7 | NA |
| Super_Scaffold_3 | 141485000 | 141505000 | bio9 | 8.62 | 2 | NA |
| Super_Scaffold_3 | 145975000 | 146035000 | bio9 | 25.47 | 1 | PRKN |
| Super_Scaffold_2 | 8405000 | 8425000 | bio9 | 10.11 | 3 | AGBL1 |
| Super_Scaffold_2 | 97285000 | 97305000 | bio9 | 16.06 | 1 | NA |
| Super_Scaffold_13 | 18555000 | 18575000 | bio9 | 12.21 | 3 | PLXDC2 |
| Super_Scaffold_13 | 51345000 | 51375000 | bio9 | 11.41 | 2 | PIK3C2G |
| Super_Scaffold_13 | 53835000 | 53855000 | bio9 | 7.99 | 1 | NA |
| Super_Scaffold_13 | 64225000 | 64245000 | bio9 | 7.85 | 1 | FGD4 |
| Super_Scaffold_13 | 68245000 | 68265000 | bio9 | 7.38 | 3 | PDZRN4 |
| Super_Scaffold_13 | 68265000 | 68285000 | bio9 | 8.79 | 2 | PDZRN4 |
| Super_Scaffold_13 | 68285000 | 68295000 | bio9 | 9.53 | 1 | PDZRN4 |
| Super_Scaffold_13 | 68295000 | 68305000 | bio9 | 9.17 | 2 | PDZRN4 |
| Super_Scaffold_13 | 68325000 | 68335000 | bio9 | 9.39 | 2 | PDZRN4 |
| Super_Scaffold_13 | 68335000 | 68345000 | bio9 | 8.10 | 1 | PDZRN4 |
| Super_Scaffold_13 | 68375000 | 68385000 | bio9 | 7.36 | 2 | PDZRN4 |
| Super_Scaffold_13 | 68385000 | 68405000 | bio9 | 9.11 | 2 | PDZRN4 |
| Super_Scaffold_13 | 68405000 | 68425000 | bio9 | 11.88 | 3 | PDZRN4 |
| Super_Scaffold_13 | 68425000 | 68445000 | bio9 | 8.23 | 2 | PDZRN4 |
| Super_Scaffold_13 | 84685000 | 84715000 | bio9 | 7.90 | 1 | NA |
| Super_Scaffold_13 | 135545000 | 135565000 | bio9 | 14.67 | 2 | SCUBE1 |
| Super_Scaffold_9 | 111305000 | 111315000 | bio9 | 7.39 | 1 | NA |
| Super_Scaffold_9 | 111315000 | 111345000 | bio9 | 10.93 | 1 | NA |
| Super_Scaffold_9 | 130845000 | 130865000 | bio9 | 10.66 | 1 | NA |
| Super_Scaffold_12 | 6335000 | 6365000 | bio9 | 10.32 | 1 | XYLB |
| Super_Scaffold_12 | 18975000 | 19015000 | bio9 | 18.83 | 3 | LOC115523127 |
| Super_Scaffold_12 | 19015000 | 19025000 | bio9 | 7.98 | 1 | LOC115523127 |
| Super_Scaffold_12 | 19025000 | 19045000 | bio9 | 10.43 | 2 | LOC115523127 |
| Super_Scaffold_12 | 19105000 | 19115000 | bio9 | 11.22 | 2 | NA |
| Super_Scaffold_12 | 123965000 | 123985000 | bio9 | 9.20 | 1 | NA |
| Super_Scaffold_14 | 5505000 | 5525000 | bio9 | 10.61 | 1 | GUCY1A2 |
| Super_Scaffold_14 | 38655000 | 38675000 | bio9 | 8.99 | 2 | SESN3 |
| Super_Scaffold_14 | 51675000 | 51695000 | bio9 | 7.27 | 2 | NA |
| Super_Scaffold_14 | 56685000 | 56725000 | bio9 | 10.43 | 1 | MAP6 |
| Super_Scaffold_14 | 75475000 | 75545000 | bio9 | 7.58 | 7 | NAV2 |
| Super_Scaffold_14 | 111825000 | 111845000 | bio9 | 7.23 | 1 | NA |
| scaffold_11_arrow_ctg1 | 3045000 | 3075000 | mean_snow_days | 7.80 | 3 | MGMT |
| scaffold_11_arrow_ctg1 | 3205000 | 3225000 | mean_snow_days | 14.21 | 1 | MGMT |
| scaffold_11_arrow_ctg1 | 3265000 | 3285000 | mean_snow_days | 7.65 | 3 | MGMT |
| scaffold_11_arrow_ctg1 | 3285000 | 3295000 | mean_snow_days | 8.17 | 1 | MGMT |
| scaffold_11_arrow_ctg1 | 3335000 | 3365000 | mean_snow_days | 8.52 | 3 | MGMT |
| scaffold_11_arrow_ctg1 | 11775000 | 11805000 | mean_snow_days | 9.17 | 1 | BAG3\|TIAL1 |
| scaffold_11_arrow_ctg1 | 11835000 | 11875000 | mean_snow_days | 9.06 | 4 | RGS10\|TIAL1 |
| scaffold_11_arrow_ctg1 | 14665000 | 14675000 | mean_snow_days | 8.76 | 1 | GFRA1 |
| scaffold_11_arrow_ctg1 | 19985000 | 19995000 | mean_snow_days | 7.38 | 1 | MXI1 |
| scaffold_11_arrow_ctg1 | 20095000 | 20115000 | mean_snow_days | 11.38 | 2 | ADD3 |
| scaffold_11_arrow_ctg1 | 40425000 | 40465000 | mean_snow_days | 9.44 | 1 | CD2H10orf71 |
| scaffold_11_arrow_ctg1 | 42535000 | 42555000 | mean_snow_days | 7.85 | 1 | BMPR1A |
| scaffold_11_arrow_ctg1 | 60905000 | 60925000 | mean_snow_days | 15.89 | 1 | LOC115527552 |
| scaffold_11_arrow_ctg1 | 60925000 | 60935000 | mean_snow_days | 16.26 | 1 | LOC115527552 |
| scaffold_11_arrow_ctg1 | 60935000 | 60945000 | mean_snow_days | 25.54 | 1 | NA |
| scaffold_11_arrow_ctg1 | 60945000 | 60955000 | mean_snow_days | 20.91 | 1 | NA |
| scaffold_11_arrow_ctg1 | 63005000 | 63025000 | mean_snow_days | 8.76 | 2 | EGR2 |
| scaffold_11_arrow_ctg1 | 63035000 | 63075000 | mean_snow_days | 14.46 | 5 | ADO\|EGR2 |
| scaffold_11_arrow_ctg1 | 63075000 | 63105000 | mean_snow_days | 27.39 | 4 | NA |
| scaffold_11_arrow_ctg1 | 63105000 | 63115000 | mean_snow_days | 13.75 | 2 | NA |
| scaffold_11_arrow_ctg1 | 63125000 | 63135000 | mean_snow_days | 8.87 | 1 | NA |
| scaffold_11_arrow_ctg1 | 63135000 | 63145000 | mean_snow_days | 8.31 | 1 | NA |
| scaffold_11_arrow_ctg1 | 68885000 | 68895000 | mean_snow_days | 16.39 | 2 | NA |
| scaffold_11_arrow_ctg1 | 73955000 | 73975000 | mean_snow_days | 8.24 | 1 | B3GALNT2 |
| scaffold_11_arrow_ctg1 | 74025000 | 74045000 | mean_snow_days | 8.08 | 2 | GNG4 |
| scaffold_11_arrow_ctg1 | 80265000 | 80285000 | mean_snow_days | 9.40 | 1 | LOC115526945 |
| scaffold_11_arrow_ctg1 | 80285000 | 80305000 | mean_snow_days | 7.72 | 1 | LOC115526945 |
| scaffold_11_arrow_ctg1 | 80635000 | 80645000 | mean_snow_days | 7.32 | 1 | A1CF |
| scaffold_11_arrow_ctg1 | 83445000 | 83475000 | mean_snow_days | 10.23 | 1 | PCDH15 |
| scaffold_11_arrow_ctg1 | 84645000 | 84665000 | mean_snow_days | 9.28 | 1 | NA |
| scaffold_11_arrow_ctg1 | 84665000 | 84705000 | mean_snow_days | 7.49 | 2 | NA |
| Super_Scaffold_6 | 1095000 | 1135000 | mean_snow_days | 9.12 | 1 | NA |
| Super_Scaffold_6 | 90425000 | 90465000 | mean_snow_days | 12.31 | 1 | TMEM132C |
| Super_Scaffold_7 | 485000 | 535000 | mean_snow_days | 12.68 | 1 | ADCK5\|DGAT1\|FBXL6\|HSF1\|SCRT1\|SLC52A2\|TMEM249 |
| Super_Scaffold_7 | 625000 | 655000 | mean_snow_days | 7.92 | 3 | HGH1\|MROH1\|WDR97 |
| Super_Scaffold_7 | 10055000 | 10075000 | mean_snow_days | 8.03 | 1 | NA |
| Super_Scaffold_7 | 37015000 | 37035000 | mean_snow_days | 10.49 | 1 | RNF19A\|SPAG1 |
| Super_Scaffold_7 | 37225000 | 37235000 | mean_snow_days | 8.59 | 1 | RGS22 |
| Super_Scaffold_7 | 38675000 | 38685000 | mean_snow_days | 10.68 | 2 | KCNS2\|STK3 |
| Super_Scaffold_7 | 45595000 | 45605000 | mean_snow_days | 15.37 | 1 | NA |
| Super_Scaffold_7 | 45605000 | 45615000 | mean_snow_days | 15.20 | 2 | NA |
| Super_Scaffold_7 | 45615000 | 45645000 | mean_snow_days | 16.52 | 3 | NA |
| Super_Scaffold_7 | 47305000 | 47335000 | mean_snow_days | 18.59 | 1 | NA |
| Super_Scaffold_7 | 74275000 | 74325000 | mean_snow_days | 9.59 | 2 | NA |
| Super_Scaffold_8 | 3375000 | 3395000 | mean_snow_days | 8.19 | 5 | NA |
| Super_Scaffold_8 | 5255000 | 5285000 | mean_snow_days | 12.94 | 2 | LOC115499820 |
| Super_Scaffold_8 | 8535000 | 8585000 | mean_snow_days | 13.61 | 1 | KCNV2 |
| Super_Scaffold_8 | 8705000 | 8725000 | mean_snow_days | 9.05 | 1 | NA |
| Super_Scaffold_8 | 16935000 | 16965000 | mean_snow_days | 13.52 | 1 | OSTF1 |
| Super_Scaffold_8 | 24205000 | 24235000 | mean_snow_days | 14.79 | 5 | FRMD3 |
| Super_Scaffold_8 | 30195000 | 30205000 | mean_snow_days | 7.74 | 1 | HSD17B3 |
| Super_Scaffold_8 | 35605000 | 35645000 | mean_snow_days | 14.53 | 1 | PTPRD |
| Super_Scaffold_8 | 43975000 | 44045000 | mean_snow_days | 12.71 | 2 | SLC24A2 |
| Super_Scaffold_8 | 65645000 | 65685000 | mean_snow_days | 7.66 | 4 | NA |
| Super_Scaffold_8 | 81315000 | 81325000 | mean_snow_days | 10.84 | 2 | LOC115499249\|PTGS1 |
| Super_Scaffold_8 | 86425000 | 86465000 | mean_snow_days | 7.31 | 1 | DPM2\|FAM102A\|PIP5KL1\|ST6GALNAC4 |
| Super_Scaffold_8 | 92085000 | 92095000 | mean_snow_days | 7.99 | 2 | CCDC187\|LHX3\|QSOX2 |
| scaffold_17_arrow_ctg1 | 10195000 | 10235000 | mean_snow_days | 7.73 | 2 | CD247\|CREG1 |
| scaffold_17_arrow_ctg1 | 10235000 | 10255000 | mean_snow_days | 7.79 | 1 | CREG1\|RCSD1 |
| scaffold_17_arrow_ctg1 | 17545000 | 17595000 | mean_snow_days | 7.81 | 1 | NA |
| scaffold_17_arrow_ctg1 | 17595000 | 17615000 | mean_snow_days | 13.74 | 1 | NA |
| scaffold_17_arrow_ctg1 | 17645000 | 17655000 | mean_snow_days | 11.63 | 1 | 1 COP |
| scaffold_17_arrow_ctg1 | 17875000 | 17885000 | mean_snow_days | 22.23 | 1 | 1 COP |
| scaffold_17_arrow_ctg1 | 17905000 | 17925000 | mean_snow_days | 24.37 | 1 | 1 COP |
| scaffold_17_arrow_ctg1 | 17985000 | 18015000 | mean_snow_days | 39.98 | 1 | NA |
| scaffold_17_arrow_ctg1 | 18035000 | 18045000 | mean_snow_days | 32.92 | 1 | NA |
| scaffold_17_arrow_ctg1 | 18115000 | 18125000 | mean_snow_days | 14.89 | 1 | NA |
| scaffold_17_arrow_ctg1 | 18405000 | 18415000 | mean_snow_days | 8.80 | 1 | PAPPA2 |
| scaffold_17_arrow_ctg1 | 46625000 | 46635000 | mean_snow_days | 13.67 | 1 | NA |
| scaffold_17_arrow_ctg1 | 46695000 | 46715000 | mean_snow_days | 12.16 | 1 | NA |
| scaffold_17_arrow_ctg1 | 55555000 | 55565000 | mean_snow_days | 11.12 | 1 | RRP15 |
| scaffold_17_arrow_ctg1 | 55895000 | 55915000 | mean_snow_days | 16.66 | 2 | NA |
| scaffold_17_arrow_ctg1 | 55915000 | 55945000 | mean_snow_days | 17.99 | 2 | NA |
| scaffold_17_arrow_ctg1 | 58935000 | 58945000 | mean_snow_days | 11.11 | 1 | NA |
| scaffold_17_arrow_ctg1 | 66025000 | 66085000 | mean_snow_days | 10.22 | 4 | KIRREL1 |
| scaffold_18_arrow_ctg1 | 5475000 | 5515000 | mean_snow_days | 11.27 | 4 | DNAH9\|SHISA6 |
| scaffold_18_arrow_ctg1 | 5555000 | 5565000 | mean_snow_days | 11.89 | 3 | DNAH9 |
| scaffold_18_arrow_ctg1 | 28655000 | 28665000 | mean_snow_days | 9.94 | 1 | MSI2 |
| scaffold_18_arrow_ctg1 | 28665000 | 28675000 | mean_snow_days | 7.53 | 2 | MSI2 |
| scaffold_18_arrow_ctg1 | 28675000 | 28685000 | mean_snow_days | 11.22 | 2 | MSI2 |
| scaffold_18_arrow_ctg1 | 54875000 | 54885000 | mean_snow_days | 10.06 | 2 | LLGL2\|LOC115501833\|LOC115501837 |
| scaffold_18_arrow_ctg1 | 54885000 | 54905000 | mean_snow_days | 16.36 | 1 | LLGL2\|LOC115501833\|LOC115501837\|RECQL5 |
| Super_Scaffold_11 | 3265000 | 3295000 | mean_snow_days | 9.15 | 1 | IRF8 |
| Super_Scaffold_11 | 3295000 | 3325000 | mean_snow_days | 11.40 | 1 | IRF8 |
| Super_Scaffold_11 | 9935000 | 9955000 | mean_snow_days | 14.78 | 2 | VAT1L |
| Super_Scaffold_11 | 15585000 | 15605000 | mean_snow_days | 9.05 | 3 | NA |
| Super_Scaffold_11 | 18805000 | 18825000 | mean_snow_days | 9.84 | 1 | AGRP\|ATP6V0D1\|HSD11B2 |
| Super_Scaffold_11 | 18905000 | 18935000 | mean_snow_days | 7.62 | 1 | KCTD19\|LRRC36 |
| Super_Scaffold_11 | 18975000 | 19005000 | mean_snow_days | 7.71 | 1 | FHOD1\|KCTD19\|LRRC29\|PLEKHG4\|SLC9A5\|TMEM208 |
| Super_Scaffold_11 | 19405000 | 19435000 | mean_snow_days | 8.53 | 4 | CMTM4\|DYNC1LI2\|TERB1 |
| Super_Scaffold_11 | 32355000 | 32365000 | mean_snow_days | 9.86 | 1 | NA |
| Super_Scaffold_11 | 40595000 | 40615000 | mean_snow_days | 9.20 | 3 | NA |
| Super_Scaffold_4 | 12825000 | 12855000 | mean_snow_days | 7.41 | 3 | IGSF21 |
| Super_Scaffold_4 | 14465000 | 14475000 | mean_snow_days | 10.04 | 2 | PLA2G2C\|UBXN10 |
| Super_Scaffold_4 | 16595000 | 16625000 | mean_snow_days | 8.25 | 1 | EPHB2 |
| Super_Scaffold_4 | 22245000 | 22275000 | mean_snow_days | 14.96 | 1 | NA |
| Super_Scaffold_4 | 22275000 | 22285000 | mean_snow_days | 11.06 | 1 | NA |
| Super_Scaffold_4 | 22285000 | 22305000 | mean_snow_days | 13.66 | 4 | NA |
| Super_Scaffold_4 | 22315000 | 22325000 | mean_snow_days | 9.44 | 1 | NA |
| Super_Scaffold_4 | 71585000 | 71605000 | mean_snow_days | 9.76 | 1 | DDAH1 |
| Super_Scaffold_4 | 73535000 | 73555000 | mean_snow_days | 9.17 | 3 | NA |
| Super_Scaffold_4 | 73565000 | 73575000 | mean_snow_days | 8.88 | 1 | NA |
| Super_Scaffold_4 | 73575000 | 73595000 | mean_snow_days | 8.47 | 1 | NA |
| Super_Scaffold_4 | 88295000 | 88305000 | mean_snow_days | 8.27 | 1 | NA |
| Super_Scaffold_4 | 93665000 | 93675000 | mean_snow_days | 17.05 | 2 | KCNC4 |
| Super_Scaffold_4 | 93705000 | 93735000 | mean_snow_days | 8.42 | 1 | NA |
| Super_Scaffold_4 | 93975000 | 93995000 | mean_snow_days | 8.54 | 1 | KCNA2 |
| Super_Scaffold_4 | 93995000 | 94005000 | mean_snow_days | 10.07 | 1 | KCNA2 |
| Super_Scaffold_4 | 117335000 | 117365000 | mean_snow_days | 7.56 | 1 | NA |
| Super_Scaffold_4 | 131155000 | 131175000 | mean_snow_days | 11.74 | 1 | LRP1B |
| Super_Scaffold_4 | 131705000 | 131755000 | mean_snow_days | 16.95 | 1 | LRP1B |
| Super_Scaffold_4 | 182935000 | 182945000 | mean_snow_days | 9.32 | 1 | ANKRD44 |
| Super_Scaffold_4 | 195285000 | 195315000 | mean_snow_days | 10.38 | 1 | NA |
| Super_Scaffold_4 | 195315000 | 195345000 | mean_snow_days | 7.90 | 1 | NA |
| Super_Scaffold_4 | 205585000 | 205605000 | mean_snow_days | 8.65 | 1 | PAX3 |
| Super_Scaffold_4 | 210005000 | 210035000 | mean_snow_days | 9.68 | 4 | AGFG1 |
| Super_Scaffold_4 | 210035000 | 210055000 | mean_snow_days | 14.64 | 2 | LOC115519919\|LOC115521792 |
| Super_Scaffold_4 | 210055000 | 210065000 | mean_snow_days | 10.14 | 3 | LOC115519919\|LOC115521792 |
| Super_Scaffold_4 | 215095000 | 215125000 | mean_snow_days | 13.92 | 2 | NA |
| Super_Scaffold_4 | 216495000 | 216505000 | mean_snow_days | 7.64 | 1 | AGAP1 |
| Super_Scaffold_5 | 1605000 | 1635000 | mean_snow_days | 11.91 | 1 | DIRAS1\|SGTA\|SLC39A3\|THOP1 |
| Super_Scaffold_5 | 29185000 | 29205000 | mean_snow_days | 16.92 | 1 | PTPRG |
| Super_Scaffold_5 | 29205000 | 29225000 | mean_snow_days | 9.73 | 2 | PTPRG |
| Super_Scaffold_5 | 31075000 | 31085000 | mean_snow_days | 10.00 | 1 | SNTN\|SYNPR |
| Super_Scaffold_5 | 32665000 | 32675000 | mean_snow_days | 7.67 | 2 | NA |
| Super_Scaffold_5 | 32685000 | 32695000 | mean_snow_days | 8.24 | 1 | NA |
| Super_Scaffold_5 | 41915000 | 41935000 | mean_snow_days | 9.70 | 2 | NA |
| Super_Scaffold_5 | 55125000 | 55155000 | mean_snow_days | 8.08 | 1 | GRIP2\|SLC6A6 |
| Super_Scaffold_5 | 110695000 | 110715000 | mean_snow_days | 7.66 | 1 | HDAC9 |
| Super_Scaffold_5 | 110715000 | 110725000 | mean_snow_days | 9.82 | 1 | HDAC9 |
| Super_Scaffold_5 | 141415000 | 141445000 | mean_snow_days | 10.79 | 2 | GPR37 |
| Super_Scaffold_5 | 165095000 | 165115000 | mean_snow_days | 8.83 | 2 | NA |
| scaffold_2_arrow_ctg1 | 11465000 | 11495000 | mean_snow_days | 8.97 | 1 | NA |
| scaffold_2_arrow_ctg1 | 32865000 | 32885000 | mean_snow_days | 9.23 | 1 | NA |
| scaffold_2_arrow_ctg1 | 33355000 | 33365000 | mean_snow_days | 9.37 | 2 | ADAMDEC1 |
| scaffold_2_arrow_ctg1 | 33365000 | 33375000 | mean_snow_days | 15.55 | 2 | ADAMDEC1 |
| scaffold_2_arrow_ctg1 | 33395000 | 33415000 | mean_snow_days | 13.54 | 3 | ADAM28\|ADAMDEC1 |
| scaffold_2_arrow_ctg1 | 56025000 | 56065000 | mean_snow_days | 11.83 | 2 | GALNTL6 |
| scaffold_2_arrow_ctg1 | 72585000 | 72605000 | mean_snow_days | 9.11 | 1 | NPY2R |
| scaffold_2_arrow_ctg1 | 72765000 | 72795000 | mean_snow_days | 7.33 | 6 | NA |
| scaffold_2_arrow_ctg1 | 100275000 | 100305000 | mean_snow_days | 12.00 | 1 | ANKRD50 |
| scaffold_2_arrow_ctg1 | 117865000 | 117875000 | mean_snow_days | 10.83 | 1 | NA |
| scaffold_2_arrow_ctg1 | 117895000 | 117905000 | mean_snow_days | 8.67 | 1 | TET2 |
| scaffold_2_arrow_ctg1 | 117905000 | 117915000 | mean_snow_days | 7.41 | 2 | TET2 |
| scaffold_2_arrow_ctg1 | 117925000 | 117955000 | mean_snow_days | 10.25 | 4 | TET2 |
| scaffold_2_arrow_ctg1 | 118005000 | 118025000 | mean_snow_days | 7.45 | 2 | TET2 |
| scaffold_2_arrow_ctg1 | 130975000 | 131015000 | mean_snow_days | 8.25 | 1 | CCSER1 |
| scaffold_2_arrow_ctg1 | 139495000 | 139515000 | mean_snow_days | 7.32 | 1 | NA |
| scaffold_2_arrow_ctg1 | 160585000 | 160615000 | mean_snow_days | 7.60 | 1 | THEGL |
| scaffold_2_arrow_ctg1 | 162035000 | 162055000 | mean_snow_days | 7.65 | 2 | NA |
| scaffold_2_arrow_ctg1 | 162205000 | 162225000 | mean_snow_days | 7.82 | 3 | KIT |
| scaffold_2_arrow_ctg1 | 162305000 | 162315000 | mean_snow_days | 7.45 | 1 | KIT |
| scaffold_2_arrow_ctg1 | 164045000 | 164075000 | mean_snow_days | 16.07 | 3 | USP46 |
| scaffold_2_arrow_ctg1 | 164075000 | 164105000 | mean_snow_days | 8.27 | 1 | USP46 |
| scaffold_2_arrow_ctg1 | 174005000 | 174025000 | mean_snow_days | 13.09 | 2 | NA |
| scaffold_2_arrow_ctg1 | 179905000 | 179915000 | mean_snow_days | 8.30 | 1 | NA |
| scaffold_2_arrow_ctg1 | 194575000 | 194615000 | mean_snow_days | 11.65 | 1 | CC2D2A\|FBXL5 |
| scaffold_2_arrow_ctg1 | 194615000 | 194625000 | mean_snow_days | 16.77 | 1 | CC2D2A\|FBXL5 |
| scaffold_2_arrow_ctg1 | 194665000 | 194695000 | mean_snow_days | 10.00 | 2 | CC2D2A |
| scaffold_2_arrow_ctg1 | 194705000 | 194745000 | mean_snow_days | 10.14 | 1 | CC2D2A |
| scaffold_2_arrow_ctg1 | 200245000 | 200265000 | mean_snow_days | 15.29 | 3 | CYTL1 |
| Super_Scaffold_1 | 6465000 | 6475000 | mean_snow_days | 8.98 | 2 | GPR12 |
| Super_Scaffold_1 | 6675000 | 6695000 | mean_snow_days | 8.69 | 2 | USP12 |
| Super_Scaffold_1 | 30895000 | 30935000 | mean_snow_days | 12.43 | 4 | NA |
| Super_Scaffold_1 | 61885000 | 61905000 | mean_snow_days | 7.41 | 1 | NA |
| Super_Scaffold_1 | 62295000 | 62325000 | mean_snow_days | 9.22 | 2 | NA |
| Super_Scaffold_1 | 62415000 | 62445000 | mean_snow_days | 9.43 | 3 | NA |
| Super_Scaffold_1 | 62465000 | 62495000 | mean_snow_days | 10.62 | 3 | NA |
| Super_Scaffold_1 | 139645000 | 139665000 | mean_snow_days | 12.97 | 1 | NA |
| Super_Scaffold_1 | 190075000 | 190125000 | mean_snow_days | 8.75 | 1 | NA |
| Super_Scaffold_1 | 195115000 | 195135000 | mean_snow_days | 13.69 | 1 | NA |
| Super_Scaffold_1 | 197735000 | 197755000 | mean_snow_days | 7.93 | 1 | NA |
| Super_Scaffold_1 | 198095000 | 198135000 | mean_snow_days | 10.84 | 3 | SH3TC2 |
| Super_Scaffold_1 | 220945000 | 220985000 | mean_snow_days | 10.25 | 2 | NA |
| Super_Scaffold_1 | 232785000 | 232825000 | mean_snow_days | 14.23 | 2 | SEMA5A |
| Super_Scaffold_3 | 1555000 | 1575000 | mean_snow_days | 8.11 | 1 | ZBED9 |
| Super_Scaffold_3 | 2965000 | 2985000 | mean_snow_days | 11.66 | 1 | ZNF184 |
| Super_Scaffold_3 | 2985000 | 3005000 | mean_snow_days | 8.84 | 1 | ZNF184 |
| Super_Scaffold_3 | 10535000 | 10545000 | mean_snow_days | 7.88 | 1 | NA |
| Super_Scaffold_3 | 12375000 | 12405000 | mean_snow_days | 12.03 | 1 | ATXN1 |
| Super_Scaffold_3 | 30235000 | 30275000 | mean_snow_days | 11.68 | 1 | NA |
| Super_Scaffold_3 | 37705000 | 37755000 | mean_snow_days | 7.49 | 1 | DNAH8 |
| Super_Scaffold_3 | 38985000 | 39005000 | mean_snow_days | 24.84 | 4 | NA |
| Super_Scaffold_3 | 39005000 | 39025000 | mean_snow_days | 22.59 | 4 | NA |
| Super_Scaffold_3 | 39025000 | 39045000 | mean_snow_days | 9.02 | 1 | LRFN2 |
| Super_Scaffold_3 | 81115000 | 81145000 | mean_snow_days | 8.95 | 1 | BACH2\|CASP8AP2\|GJA10 |
| Super_Scaffold_3 | 81255000 | 81275000 | mean_snow_days | 12.60 | 2 | BACH2 |
| Super_Scaffold_3 | 86785000 | 86795000 | mean_snow_days | 9.41 | 1 | NA |
| Super_Scaffold_3 | 140845000 | 140865000 | mean_snow_days | 14.00 | 1 | NA |
| Super_Scaffold_3 | 141465000 | 141495000 | mean_snow_days | 8.92 | 2 | NA |
| Super_Scaffold_3 | 151685000 | 151695000 | mean_snow_days | 7.72 | 1 | THBS2 |
| Super_Scaffold_2 | 8405000 | 8435000 | mean_snow_days | 14.73 | 3 | AGBL1 |
| Super_Scaffold_2 | 13355000 | 13375000 | mean_snow_days | 7.46 | 3 | NA |
| Super_Scaffold_2 | 13615000 | 13655000 | mean_snow_days | 7.62 | 1 | NA |
| Super_Scaffold_2 | 69855000 | 69875000 | mean_snow_days | 8.46 | 2 | LOC115516166\|LOC115517158\|LOC115517160 |
| Super_Scaffold_2 | 76115000 | 76155000 | mean_snow_days | 7.93 | 1 | NA |
| Super_Scaffold_13 | 18555000 | 18575000 | mean_snow_days | 11.44 | 3 | PLXDC2 |
| Super_Scaffold_13 | 38595000 | 38625000 | mean_snow_days | 9.51 | 2 | CRACR2A |
| Super_Scaffold_13 | 51315000 | 51335000 | mean_snow_days | 7.70 | 2 | PIK3C2G |
| Super_Scaffold_13 | 64235000 | 64255000 | mean_snow_days | 7.32 | 1 | FGD4 |
| Super_Scaffold_13 | 67495000 | 67525000 | mean_snow_days | 15.37 | 1 | LOC115518678\|MUC19 |
| Super_Scaffold_13 | 67535000 | 67565000 | mean_snow_days | 7.77 | 1 | MUC19 |
| Super_Scaffold_13 | 68235000 | 68265000 | mean_snow_days | 25.76 | 3 | PDZRN4 |
| Super_Scaffold_13 | 68265000 | 68285000 | mean_snow_days | 31.08 | 2 | PDZRN4 |
| Super_Scaffold_13 | 68285000 | 68295000 | mean_snow_days | 35.59 | 1 | PDZRN4 |
| Super_Scaffold_13 | 68295000 | 68305000 | mean_snow_days | 33.86 | 2 | PDZRN4 |
| Super_Scaffold_13 | 68305000 | 68335000 | mean_snow_days | 46.29 | 4 | PDZRN4 |
| Super_Scaffold_13 | 68335000 | 68345000 | mean_snow_days | 34.18 | 1 | PDZRN4 |
| Super_Scaffold_13 | 68345000 | 68365000 | mean_snow_days | 20.21 | 3 | PDZRN4 |
| Super_Scaffold_13 | 68365000 | 68375000 | mean_snow_days | 11.19 | 1 | PDZRN4 |
| Super_Scaffold_13 | 68375000 | 68385000 | mean_snow_days | 31.32 | 2 | PDZRN4 |
| Super_Scaffold_13 | 68385000 | 68395000 | mean_snow_days | 19.01 | 1 | PDZRN4 |
| Super_Scaffold_13 | 68395000 | 68405000 | mean_snow_days | 31.93 | 1 | PDZRN4 |
| Super_Scaffold_13 | 68405000 | 68415000 | mean_snow_days | 25.86 | 1 | PDZRN4 |
| Super_Scaffold_13 | 68415000 | 68425000 | mean_snow_days | 37.90 | 2 | PDZRN4 |
| Super_Scaffold_13 | 68425000 | 68445000 | mean_snow_days | 31.35 | 2 | PDZRN4 |
| Super_Scaffold_13 | 68445000 | 68455000 | mean_snow_days | 12.17 | 2 | PDZRN4 |
| Super_Scaffold_13 | 68455000 | 68465000 | mean_snow_days | 11.42 | 1 | PDZRN4 |
| Super_Scaffold_13 | 68465000 | 68475000 | mean_snow_days | 15.80 | 3 | PDZRN4 |
| Super_Scaffold_13 | 75205000 | 75225000 | mean_snow_days | 7.36 | 3 | CCNT1\|TEX49 |
| Super_Scaffold_13 | 76595000 | 76635000 | mean_snow_days | 7.97 | 1 | FAM186A |
| Super_Scaffold_13 | 77855000 | 77885000 | mean_snow_days | 7.88 | 2 | FIGNL2\|LOC115518703\|SCN8A |
| Super_Scaffold_13 | 80515000 | 80535000 | mean_snow_days | 10.94 | 9 | TESPA1 |
| Super_Scaffold_13 | 84685000 | 84695000 | mean_snow_days | 7.77 | 1 | NA |
| Super_Scaffold_13 | 105375000 | 105425000 | mean_snow_days | 12.54 | 3 | TMTC2 |
| Super_Scaffold_9 | 2295000 | 2325000 | mean_snow_days | 7.80 | 2 | CDH4 |
| Super_Scaffold_9 | 2795000 | 2815000 | mean_snow_days | 8.04 | 1 | NA |
| Super_Scaffold_9 | 2815000 | 2825000 | mean_snow_days | 7.34 | 3 | NA |
| Super_Scaffold_9 | 4365000 | 4385000 | mean_snow_days | 7.45 | 2 | NPEPL1 |
| Super_Scaffold_9 | 5295000 | 5315000 | mean_snow_days | 11.75 | 3 | PMEPA1\|ZBP1 |
| Super_Scaffold_9 | 5865000 | 5885000 | mean_snow_days | 12.19 | 4 | NA |
| Super_Scaffold_9 | 15215000 | 15245000 | mean_snow_days | 7.84 | 1 | LOC115509630\|SLPI |
| Super_Scaffold_9 | 45775000 | 45785000 | mean_snow_days | 8.56 | 1 | KIZ |
| Super_Scaffold_9 | 46175000 | 46215000 | mean_snow_days | 12.13 | 3 |  |
| Super_Scaffold_9 | 130845000 | 130865000 | mean_snow_days | 15.01 | 1 | NA |
| Super_Scaffold_12 | 6335000 | 6365000 | mean_snow_days | 8.48 | 1 | XYLB |
| Super_Scaffold_12 | 6855000 | 6875000 | mean_snow_days | 13.09 | 1 | SCN11A |
| Super_Scaffold_12 | 18975000 | 18995000 | mean_snow_days | 7.51 | 1 | LOC115523127 |
| Super_Scaffold_12 | 18995000 | 19005000 | mean_snow_days | 21.25 | 2 | LOC115523127 |
| Super_Scaffold_12 | 19005000 | 19025000 | mean_snow_days | 23.50 | 1 | LOC115523127 |
| Super_Scaffold_12 | 19025000 | 19045000 | mean_snow_days | 13.76 | 2 | LOC115523127 |
| Super_Scaffold_12 | 19055000 | 19065000 | mean_snow_days | 10.34 | 2 | LOC115523127 |
| Super_Scaffold_12 | 19065000 | 19075000 | mean_snow_days | 9.66 | 2 | LOC115523127 |
| Super_Scaffold_12 | 19075000 | 19085000 | mean_snow_days | 9.86 | 2 | LOC115523127 |
| Super_Scaffold_12 | 19085000 | 19105000 | mean_snow_days | 11.31 | 3 | LOC115523127 |
| Super_Scaffold_12 | 19105000 | 19115000 | mean_snow_days | 15.36 | 2 | NA |
| Super_Scaffold_12 | 35815000 | 35835000 | mean_snow_days | 8.15 | 1 | CLSTN2 |
| Super_Scaffold_12 | 35845000 | 35865000 | mean_snow_days | 10.96 | 1 | CLSTN2 |
| Super_Scaffold_12 | 50105000 | 50135000 | mean_snow_days | 14.35 | 3 | NA |
| Super_Scaffold_12 | 84195000 | 84205000 | mean_snow_days | 8.03 | 1 | NA |
| Super_Scaffold_12 | 84465000 | 84485000 | mean_snow_days | 7.94 | 3 | NA |
| Super_Scaffold_12 | 120635000 | 120655000 | mean_snow_days | 9.07 | 2 | CADM2 |
| Super_Scaffold_12 | 123955000 | 123975000 | mean_snow_days | 12.63 | 1 | NA |
| Super_Scaffold_12 | 124065000 | 124095000 | mean_snow_days | 11.18 | 4 | NA |
| Super_Scaffold_12 | 149985000 | 150005000 | mean_snow_days | 7.83 | 1 | CLDN14 |
| Super_Scaffold_12 | 154105000 | 154135000 | mean_snow_days | 14.43 | 2 | NA |
| Super_Scaffold_14 | 5505000 | 5525000 | mean_snow_days | 12.27 | 1 | GUCY1A2 |
| Super_Scaffold_14 | 24145000 | 24175000 | mean_snow_days | 9.33 | 3 | NA |
| Super_Scaffold_14 | 38655000 | 38665000 | mean_snow_days | 8.10 | 1 | SESN3 |
| Super_Scaffold_14 | 38665000 | 38695000 | mean_snow_days | 11.46 | 1 | SESN3 |
| Super_Scaffold_14 | 56695000 | 56715000 | mean_snow_days | 11.81 | 1 | MAP6 |
| Super_Scaffold_14 | 67765000 | 67795000 | mean_snow_days | 11.34 | 1 | NA |
| Super_Scaffold_14 | 67905000 | 67935000 | mean_snow_days | 12.24 | 6 | GALNT18 |
| Super_Scaffold_14 | 74605000 | 74615000 | mean_snow_days | 7.90 | 2 | LOC115525771\|TSG101 |
| Super_Scaffold_14 | 75535000 | 75545000 | mean_snow_days | 20.30 | 2 | NAV2 |
| Super_Scaffold_14 | 75545000 | 75565000 | mean_snow_days | 8.90 | 2 | NAV2 |
| Super_Scaffold_14 | 82855000 | 82865000 | mean_snow_days | 10.15 | 1 | KIF18A |
| Super_Scaffold_14 | 82875000 | 82895000 | mean_snow_days | 8.32 | 3 | KIF18A |
| Super_Scaffold_14 | 94265000 | 94295000 | mean_snow_days | 7.62 | 3 | NA |
| Super_Scaffold_14 | 94295000 | 94305000 | mean_snow_days | 9.29 | 1 | NA |
| Super_Scaffold_14 | 94305000 | 94345000 | mean_snow_days | 8.09 | 3 | NA |
| Super_Scaffold_14 | 97765000 | 97785000 | mean_snow_days | 8.62 | 4 | PRDM11 |
| Super_Scaffold_14 | 100085000 | 100115000 | mean_snow_days | 8.72 | 2 | NUP160 |
| Super_Scaffold_14 | 104825000 | 104845000 | mean_snow_days | 21.98 | 2 | LOC115525320\|LOC115526000\|LOC115526443 |
| Super_Scaffold_14 | 104935000 | 104945000 | mean_snow_days | 13.36 | 2 | LOC115526000\|MS4A14\|MS4A5 |
| Super_Scaffold_14 | 104945000 | 104965000 | mean_snow_days | 9.65 | 4 | LOC115526000\|MS4A14\|MS4A1\|MS4A5 |
| Super_Scaffold_14 | 108465000 | 108505000 | mean_snow_days | 7.66 | 1 | ARL2\|BATF2\|LOC115524781\|NAALADL1\|SAC3D1\|SNX15\|TMEM262\|ZFPL1 |
| Super_Scaffold_14 | 113325000 | 113345000 | mean_snow_days | 8.73 | 1 | LOC115524804\|MRGPRG\|NADSYN1 |
| Super_Scaffold_14 | 115025000 | 115065000 | mean_snow_days | 8.00 | 1 | IFITM5\|LOC115525095\|LOC115525096\|LOC115525097\|NLRP6\|PGGHG |

**Table S7.** Rotation values of the different environmental predictors in the PCA conducted on the GDM transformed raster layers using neutral loci.

| **Predictor** | **PC1** | **PC2** | **PC3** |
| --- | --- | --- | --- |
| **x-coord** | **0.999** | -0.003 | -0.03 |
| **y-coord** | -0.023 | **-0.544** | **-0.498** |
| **T_mean_year** | 0.001 | 0.141 | 0.048 |
| **T_range_day** | 0.003 | 0.062 | -0.008 |
| **Iso_T** | -0.02 | **0.799** | **-0.392** |
| **T_max_warm** | 0.012 | 0.114 | 0.22 |
| **T_warm_quart** | 0.002 | 0.059 | 0.035 |
| **P_seasonality** | 0.026 | 0.158 | 0.039 |
| **P_warm_quart** | 0.009 | 0.004 | **0.737** |

**Table S8.** Rotation values of the different environmental predictors in the PCA conducted on the GDM transformed raster layers using candidate loci.

| **Predictor** | **PC1** | **PC2** | **PC3** |
| --- | --- | --- | --- |
| **T_range_day** | **0.916** | 0.401 | 0.005 |
| **Jan_mean_depth** | -0.082 | 0.175 | **0.981** |
| **Mean_snow_days** | -0.393 | **0.899** | -0.193 |

###

### Supplementary Figures


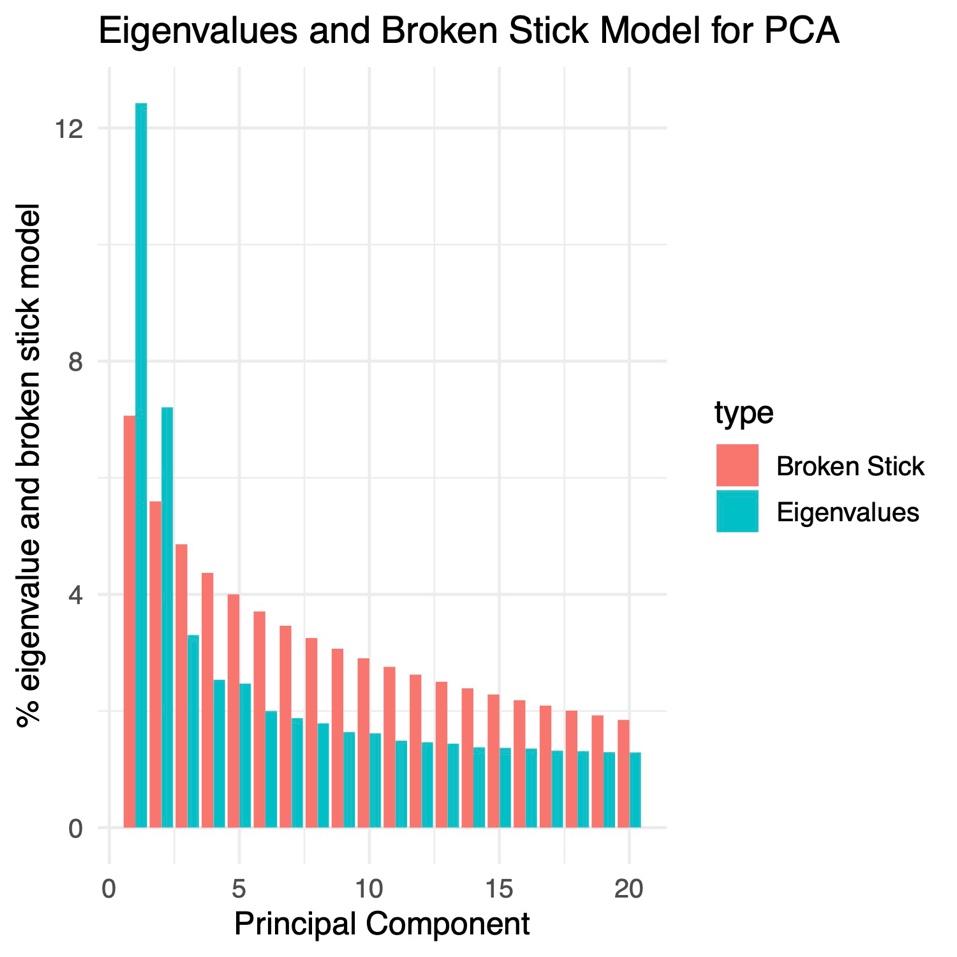


**Figure S1.** Comparison of eigenvalues percentages of the neutral PCA to a broken stick model.


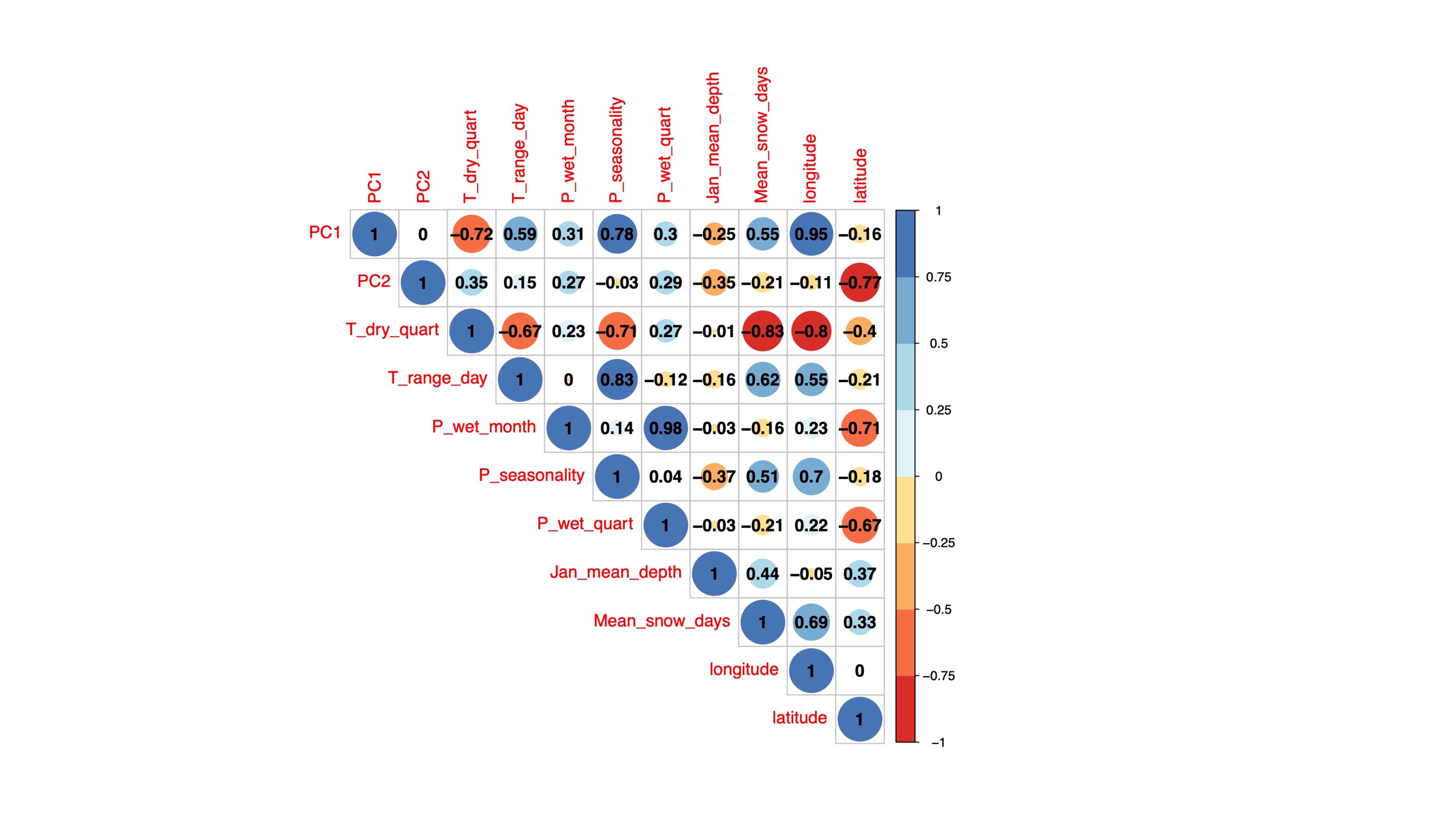


**Figure S2.** Correlogram of the scores of the first two principal components of a neutral PCA, environmental predictors and geographic locations.


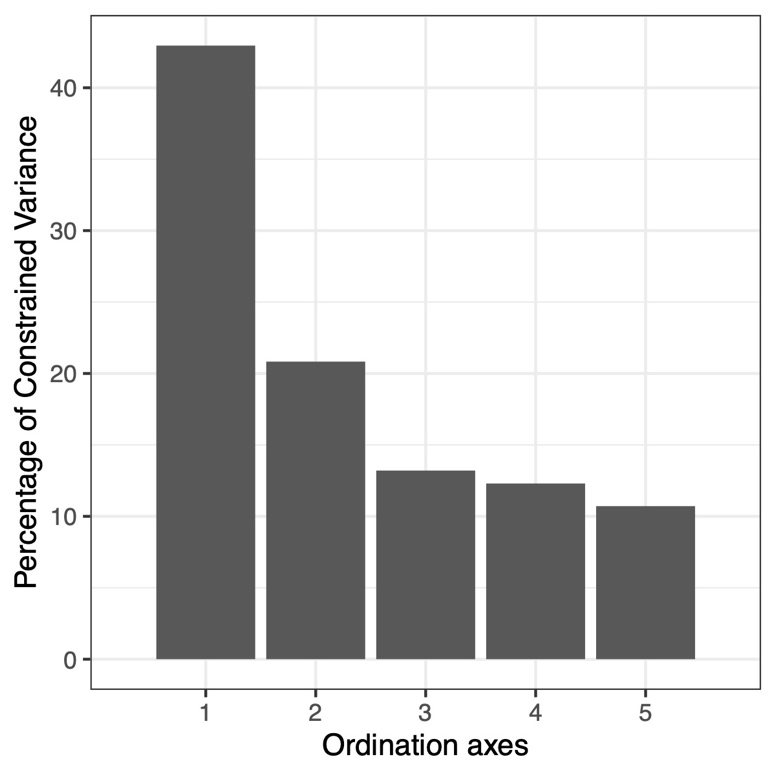


**Figure S3.** Scree plot representing the amount of variance explained by each axis of the redundancy analysis, including the 5 selected environmental predictors and the pruned SNPs dataset.


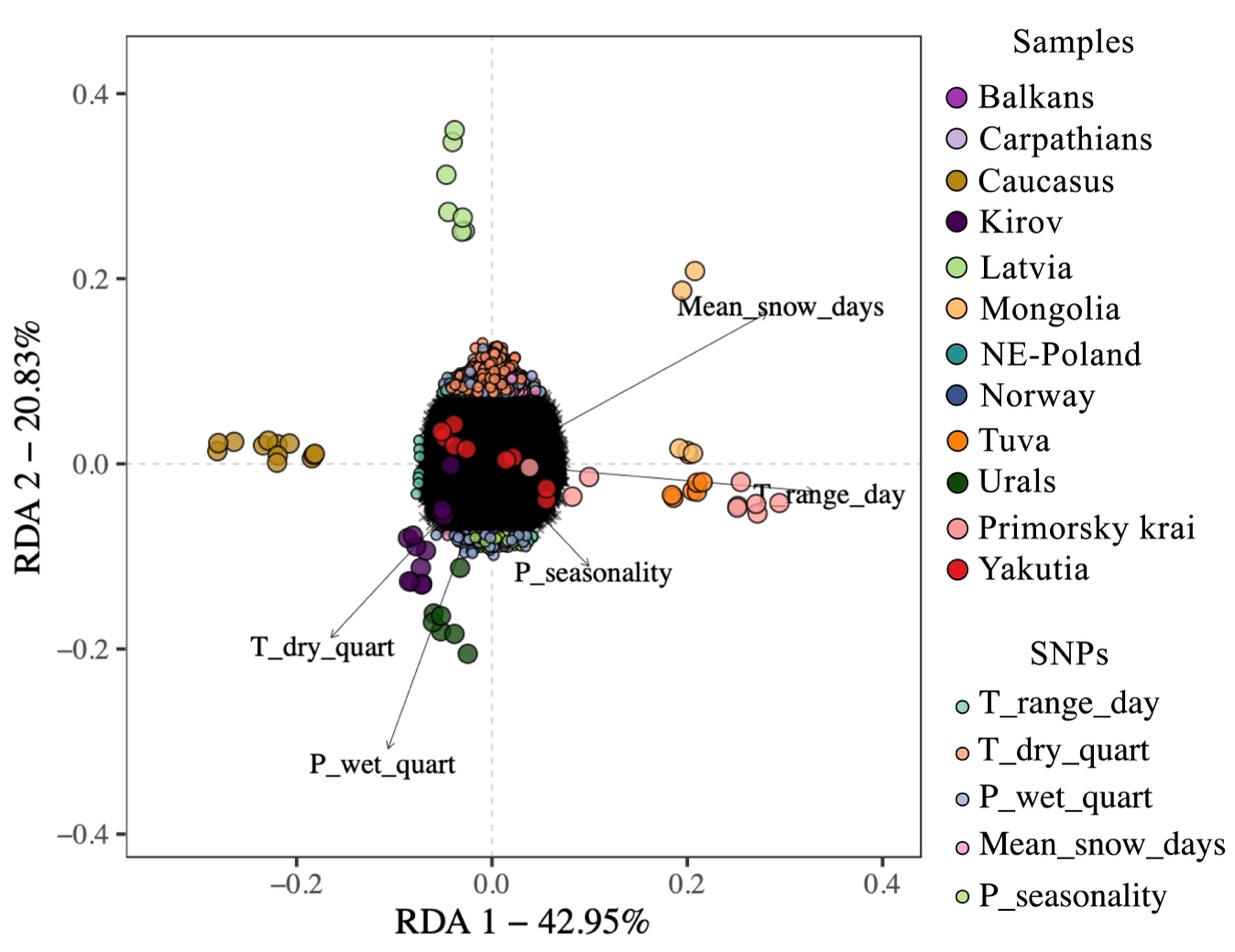


**Figure S4.** SNP and sample loadings on RDA axes 1 and 2. Non-significant SNPs are represented by a dark grey cross. Significant SNPs (small circles) are color coded to reflect which predictor variable (vector lines) they most strongly correlate with. Samples (big circles) are color-coded by their population.


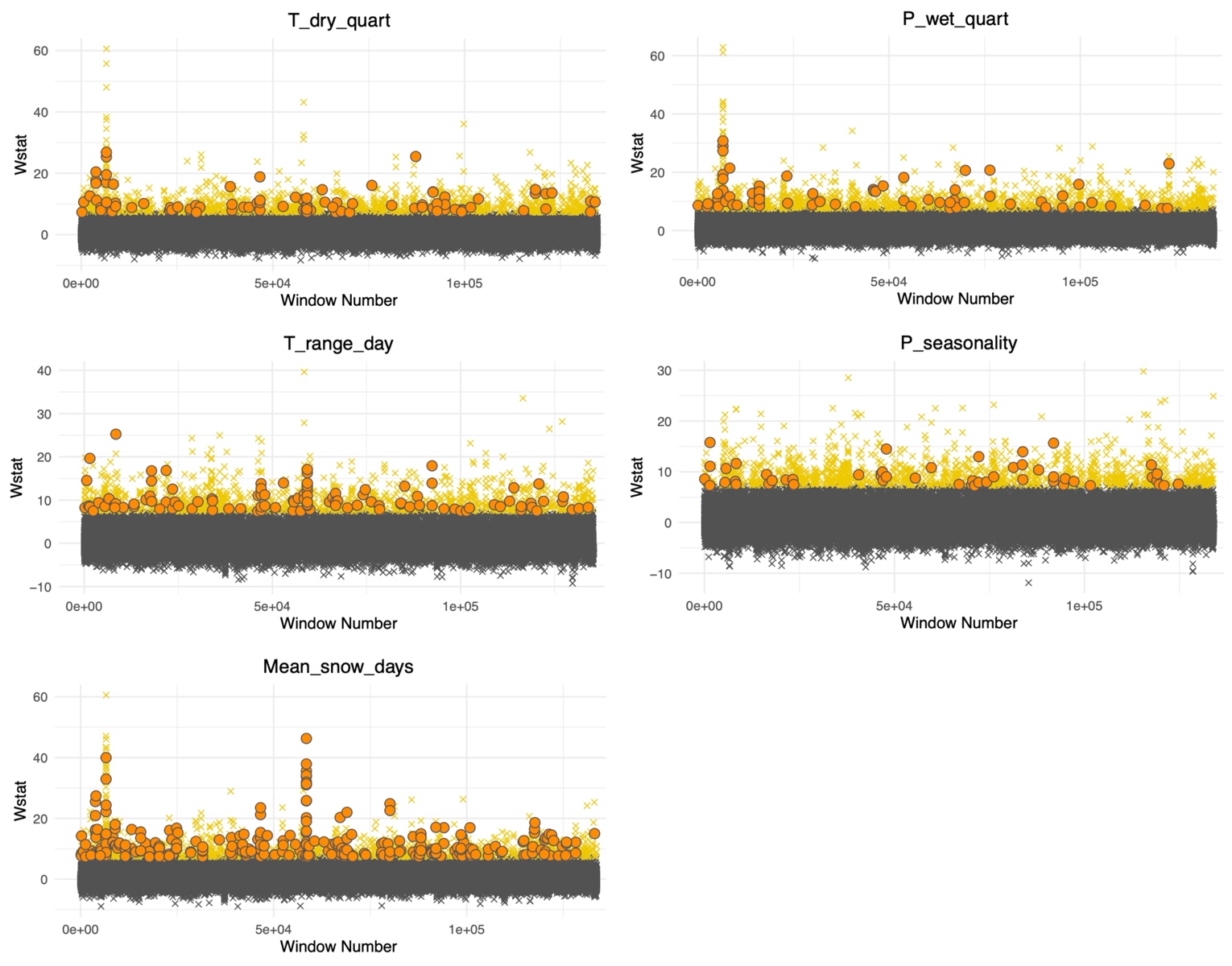


**Figure S5.** Manhattan plots showing the results of the intersection of multivariate (RDA) and genomic windows delimited by GenWin, based on individual (BayPass) analyses of each of the five environmental predictors. Non-candidate windows are represented by gray crosses. Outlier windows (>99 percentile of Wstat) are represented by yellow crosses. Outlier windows that overlap with at least one candidate SNP from the multivariate analysis are represented by a yellow circle.


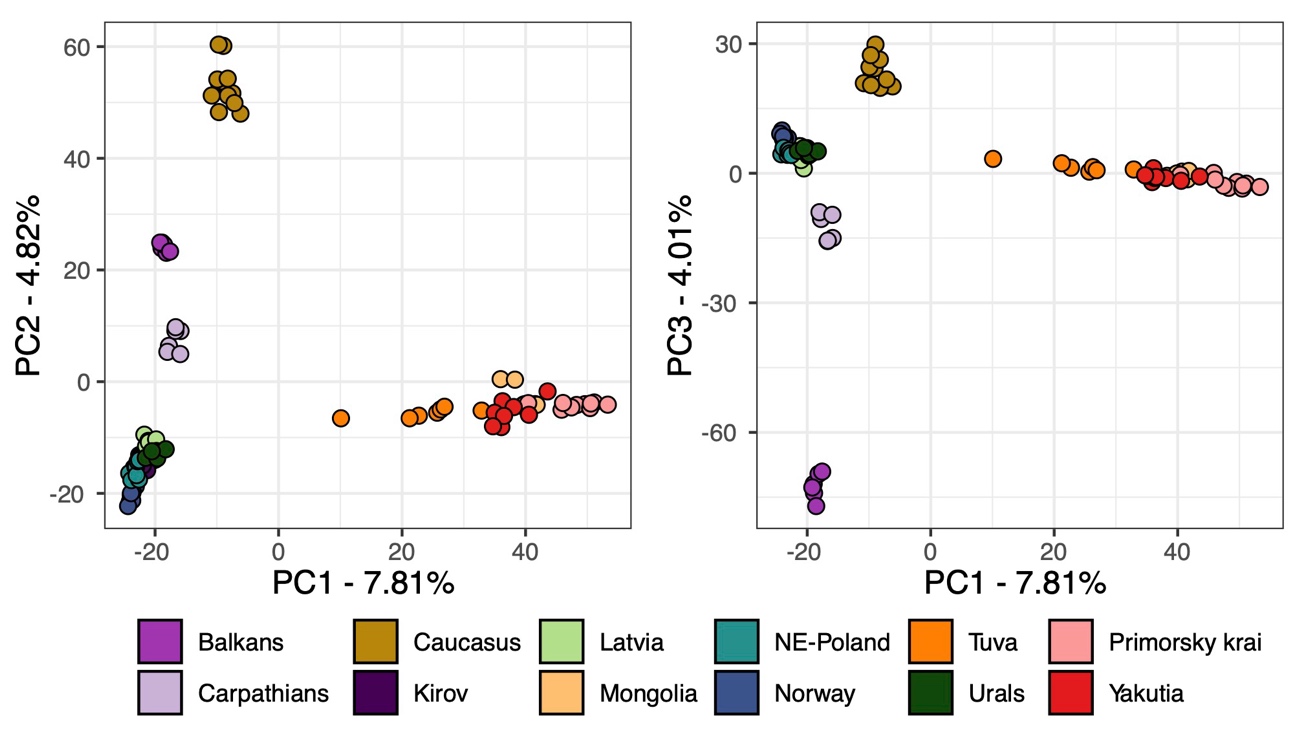


**Figure S6.** Representation of neutral PCA, with PC1 and PC2 on the left and PC1 and PC3 on the right. Samples are color-coded by population.


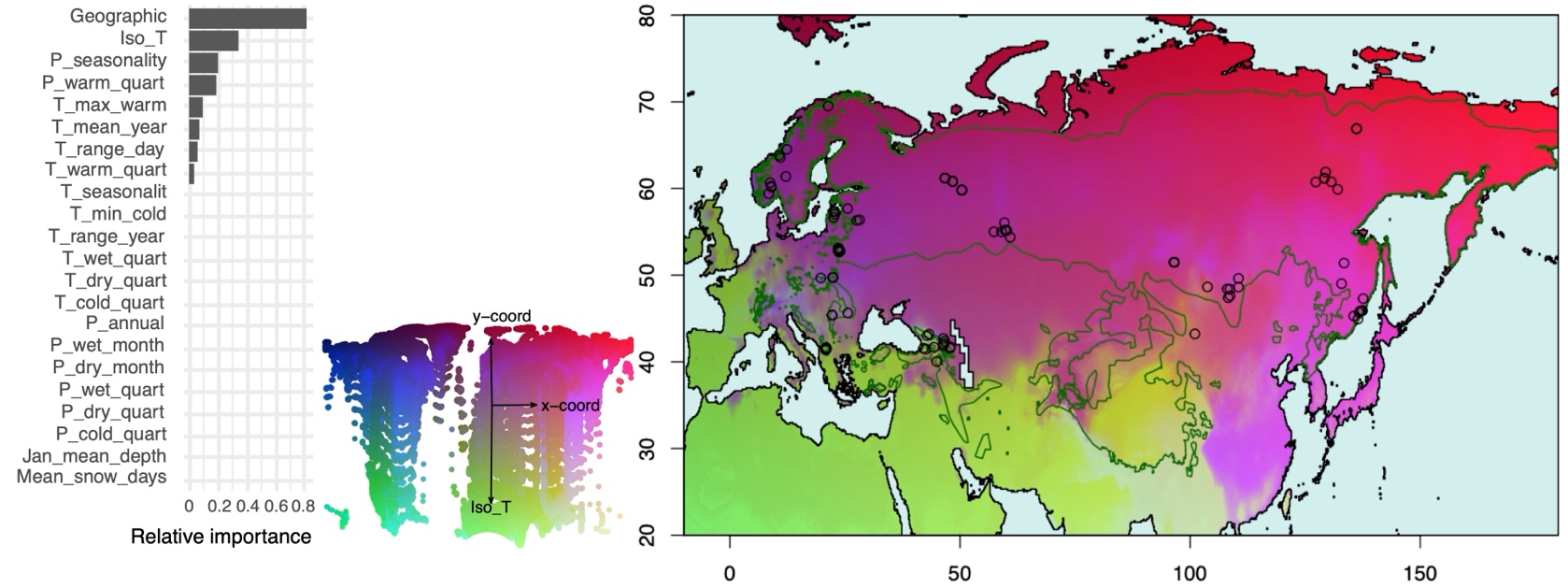


**Figure S7** Predicted turnover in neutral genetic composition as calculated by GDM (right) as consequence of gradients of the different environmental predictors (left). Locations with similar colors are expected to have similar genetic composition, based on the effects of the first three principal component gradients (biplot in middle). Circles represent sampled individuals and green line delimits Eurasian lynx current distributional range (Breitenmoser, Breitenmoser-Würsten, Lanz, von Arx, & Antonevich, 2015).


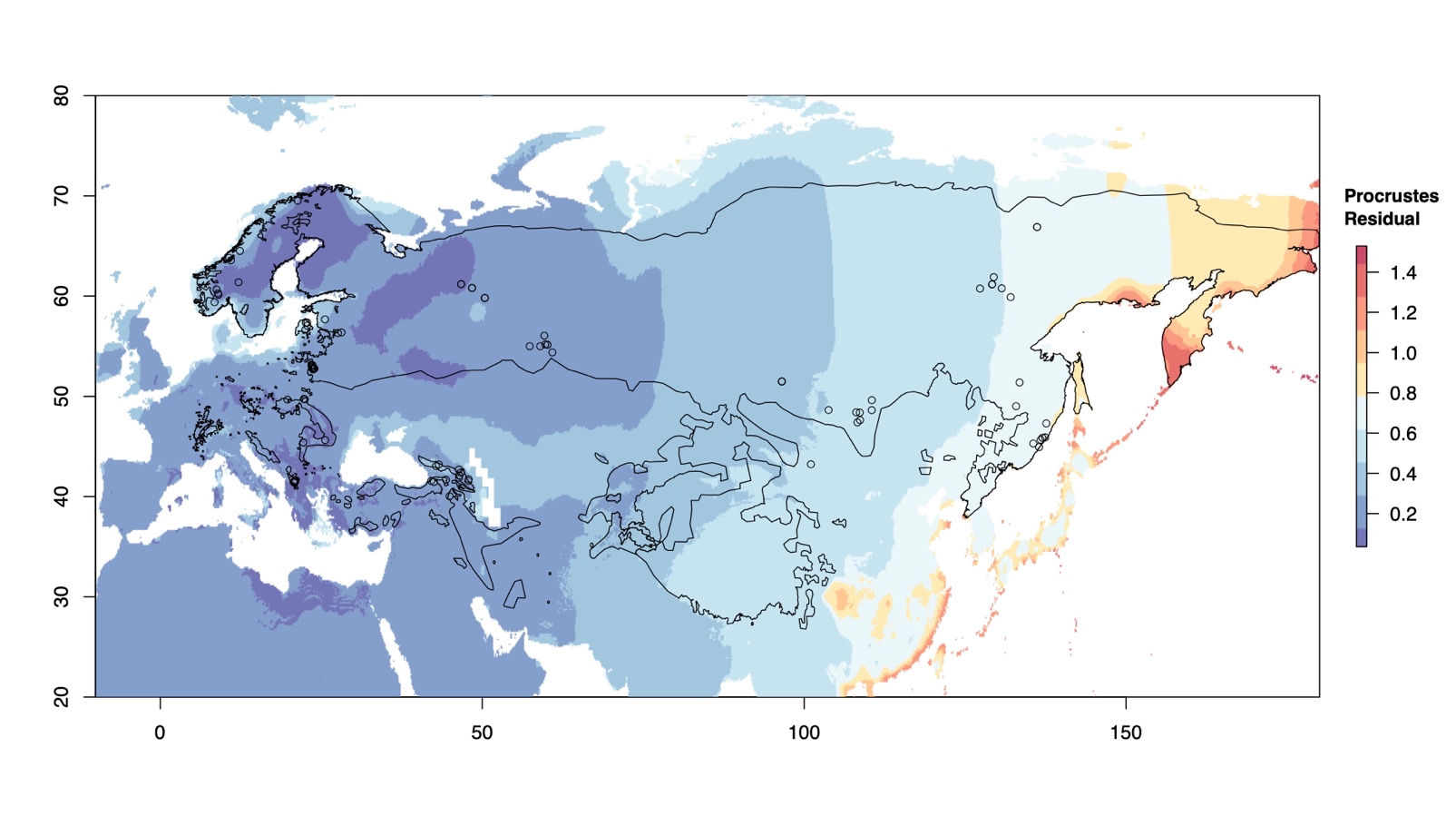


**Figure S8.** Estimated difference between the turnover rates in neutral and adaptive genetic composition based on residuals of Procrustes superimposition between the two. Circles represent sampled individuals and black line delimits Eurasian lynx current distributional range (Breitenmoser, Breitenmoser-Würsten, Lanz, von Arx, & Antonevich, 2015).
